# Change in cardiac output during Trendelenburg maneuver is a reliable predictor of fluid responsiveness in patients with acute respiratory distress syndrome in the prone position under protective ventilation

**DOI:** 10.1186/s13054-017-1881-0

**Published:** 2017-12-05

**Authors:** Hodane Yonis, Laurent Bitker, Mylène Aublanc, Sophie Perinel Ragey, Zakaria Riad, Floriane Lissonde, Aurore Louf-Durier, Sophie Debord, Florent Gobert, Romain Tapponnier, Claude Guérin, Jean-Christophe Richard

**Affiliations:** 1 Service de Réanimation Médicale, Hôpital De La Croix Rousse, Hospices Civils de Lyon, 103 Grande Rue de la Croix Rousse, 69004 Lyon, France; 2 Université de Lyon, Université LYON I, Lyon, France; 3 IMRB, INSERM 955Eq13, Créteil, France; 4 CREATIS INSERM 1044 CNRS 5220, Villeurbanne, France

**Keywords:** Acute respiratory distress syndrome, Prone position, Cardiac output, Acute circulatory failure, Fluid responsiveness, Protective ventilation

## Abstract

**Background:**

Predicting fluid responsiveness may help to avoid unnecessary fluid administration during acute respiratory distress syndrome (ARDS). The aim of this study was to evaluate the diagnostic performance of the following methods to predict fluid responsiveness in ARDS patients under protective ventilation in the prone position: cardiac index variation during a Trendelenburg maneuver, cardiac index variation during an end-expiratory occlusion test, and both pulse pressure variation and change in pulse pressure variation from baseline during a tidal volume challenge by increasing tidal volume (VT) to 8 ml.kg^-1^.

**Methods:**

This study is a prospective single-center study, performed in a medical intensive care unit, on ARDS patients with acute circulatory failure in the prone position. Patients were studied at baseline, during a 1-min shift to the Trendelenburg position, during a 15-s end-expiratory occlusion, during a 1-min increase in VT to 8 ml.kg^-1^, and after fluid administration. Fluid responsiveness was deemed present if cardiac index assessed by transpulmonary thermodilution increased by at least 15% after fluid administration.

**Results:**

There were 33 patients included, among whom 14 (42%) exhibited cardiac arrhythmia at baseline and 15 (45%) were deemed fluid-responsive. The area under the receiver operating characteristic (ROC) curve of the pulse contour-derived cardiac index change during the Trendelenburg maneuver and the end-expiratory occlusion test were 0.90 (95% CI, 0.80–1.00) and 0.65 (95% CI, 0.46–0.84), respectively. An increase in cardiac index ≥ 8% during the Trendelenburg maneuver enabled diagnosis of fluid responsiveness with sensitivity of 87% (95% CI, 67–100), and specificity of 89% (95% CI, 72–100). The area under the ROC curve of pulse pressure variation and change in pulse pressure variation during the tidal volume challenge were 0.52 (95% CI, 0.24–0.80) and 0.59 (95% CI, 0.31–0.88), respectively.

**Conclusions:**

Change in cardiac index during a Trendelenburg maneuver is a reliable test to predict fluid responsiveness in ARDS patients in the prone position, while neither change in cardiac index during end-expiratory occlusion, nor pulse pressure variation during a VT challenge reached acceptable predictive performance to predict fluid responsiveness in this setting.

**Trial registration:**

ClinicalTrials.gov, NCT01965574. Registered on 16 October 2013. The trial was registered 6 days after inclusion of the first patient.

**Electronic supplementary material:**

The online version of this article (doi:10.1186/s13054-017-1881-0) contains supplementary material, which is available to authorized users.

## Background

Predicting fluid responsiveness is of paramount importance to avoid unnecessary fluid administration in patients with acute respiratory distress syndrome (ARDS), since a positive fluid balance is strongly associated with ARDS mortality [1, 2]. Several tests with high reliability in prediction of fluid responsiveness may help optimization of fluid administration to achieve a neutral or negative fluid balance in this condition.

Pulse pressure variation (PPV) [3–5] and other related tests exploring intra-tidal cyclic changes in hemodynamics during mechanical ventilation [6–9] are highly reliable to detect fluid responsiveness, as long as the tidal volume (VT) is greater than 8 ml.kg^-1^, the cardiac rhythm is regular, the ratio of heart rate to respiratory rate remains high [10], and both compliance of the respiratory system and abdominal pressure stay in the normal range. However, all these validity criteria are strongly challenged in patients with ARDS under protective ventilation [11–13], even more so in the prone position (PP).

Cardiac index variation during an end-expiratory occlusion (EEO), by transiently suppressing cardiopulmonary interaction, and hence the cyclic impediment to cardiac preload during inspiration, is reliable in supine patients with ARDS to detect fluid responsiveness [14], but has been validated with VT slightly higher than 6 ml.kg^-1^. Since low respiratory system compliance decreases airway pressure transmission to intravascular pressure [15], the validity of this test may be challenged in patients with severe ARDS under protective ventilation (VT of 6 ml.kg^-1^ predicted body weight (PBW) or lower).

Cardiac index variation during passive leg raising is also a reliable method to identify fluid responsiveness [16], free of the limitations of the previously described tests, but is impracticable in the PP. The Trendelenburg maneuver may be an interesting alternative to transiently modify cardiac preload, and identify fluid responsiveness. None of the previous tests have been validated in the PP in patients with ARDS, although this treatment is now a therapeutic standard in severe ARDS [17].

## Methods

### Study aim

The primary aim of this study was to evaluate the diagnostic performance of cardiac index variation during a Trendelenburg maneuver to predict fluid responsiveness in patients with ARDS under protective ventilation in the PP. Secondary objectives were to evaluate the diagnostic performance of cardiac index variation during an EEO, and both PPV and change in PPV from baseline during a VT challenge from 6 to 8 ml.kg^-1^ PBW.

### Study design

This study is a prospective single-center study, performed between October 2013 and January 2017 in a 15-bed medical ICU and registered at ClinicalTrials.gov (NCT01965574). The study protocol (see Additional file 1) was approved by the local ethics committee (Comité de Protection des Personnes Sud-Est IV, ID-RCB-2013-A00526-39). Written consent from the patients’ closest relatives was required for inclusion, and eventually confirmed by the patient after ARDS resolution.

### Patients

The subjects had to fulfill all the following inclusion criteria: ARDS according to the Berlin definition [18], ongoing session of PP under invasive mechanical ventilation, ongoing monitoring with the PiCCO® device (Pulsion Medical Systems, Feldkirchen, Germany), and decision by the attending physician to administer fluids with at least one criterion of acute circulatory failure among the following: arterial lactate >2 mmol.L^-1^, mean arterial pressure <65 mm Hg, cardiac output decrease, urine output <0.5 ml.kg.h^-1^, heart rate >100 min^-1^ and skin mottling.

Non-inclusion criteria were the following: age <18 years, contra-indication to the Trendelenburg position, pregnancy, lower limbs amputation, known obstruction of inferior vena cava, previous inclusion in current study, and patient under a legal protection measure as required by French regulation. Patients exhibiting respiratory effort detected on the pressure-time curve displayed on the ventilator during a 15-s EEO were excluded from the study.

### Protocol description

**Fig. 1:**
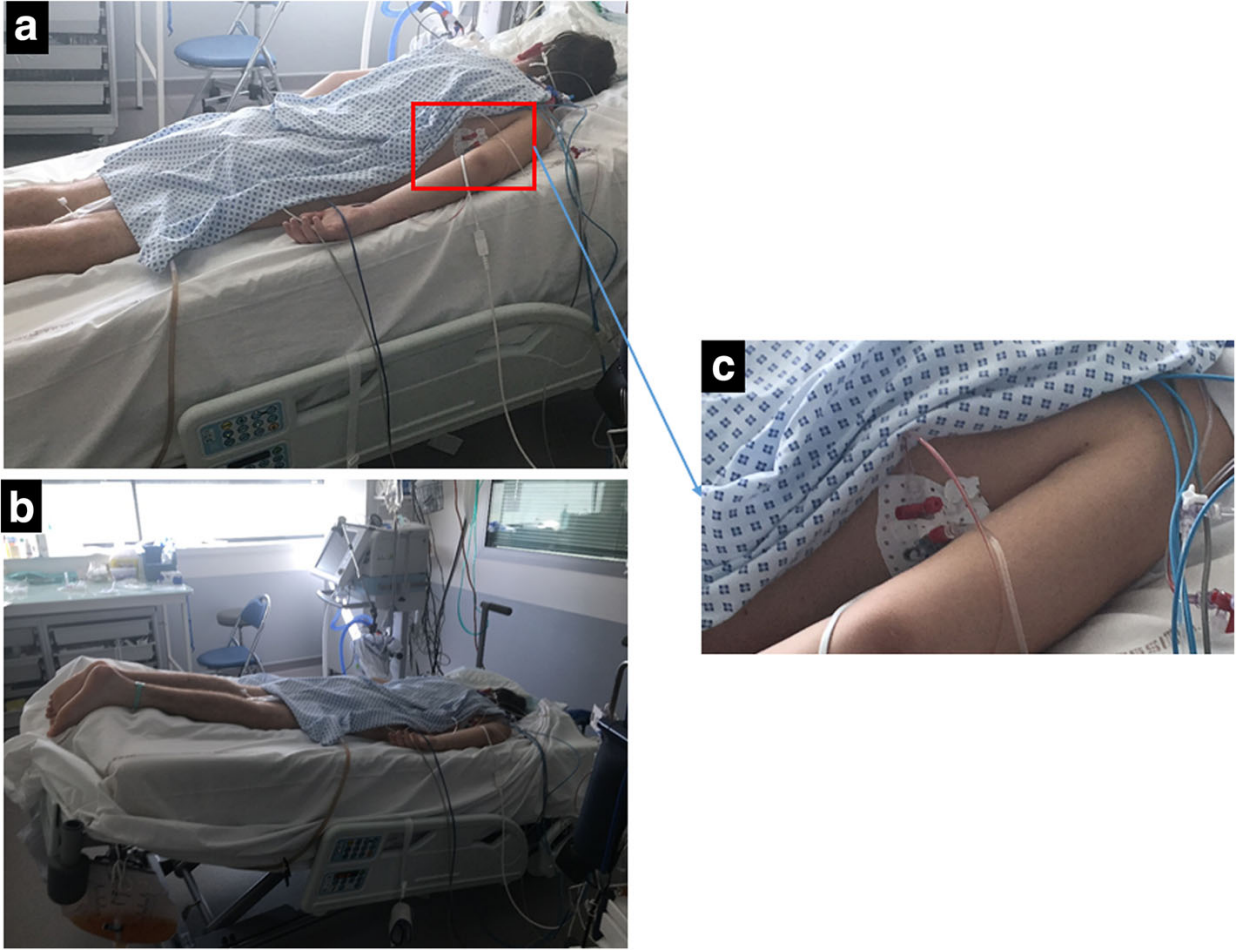
Trendelenburg maneuver. **a** Starting position of the Trendelenburg maneuver with bed angulation +13°. **b** Trendelenburg position with a −13° downward bed angulation. **c** Pressure transducers taped on the thorax at the phlebostatic reference point

Patients were deeply sedated with a combination of morphine and midazolam targeting a Ramsay score of 6 [19], and remained in PP with a 13° upward bed angulation throughout the study, except during the Trendelenburg maneuver. They were ventilated in volume-controlled mode with a VT 6 ml.kg^-1^ PBW. Patients were studied at baseline (baseline-1), during a 1-min postural change to the Trendelenburg position with a −13° downward bed angulation (Fig. 1), during a 1-min VT challenge at 8 ml.kg^-1^ PBW, during a 15-s EEO maneuver, and after intravenous infusion (IV) of 500 ml crystalloids over 15 min. Patients were returned to baseline settings for 1 min after each intervention (see Additional file 2). The following adverse events were prospectively collected throughout the protocol: drop of systolic arterial pressure >30 mm Hg, increase in heart rate >10%, decrease in peripheral oxygen saturation <88%, new onset of cardiac arrhythmia, or any other adverse event considered relevant by investigators.

### Measurements

Jugular central venous and femoral arterial lines were connected to an Intellivue MP40 monitor equipped with a PiCCO® module (Philips Healthcare, Andover, MA, USA). Pressure transducers were taped on the thorax at the phlebostatic reference point (Fig. 1). The following hemodynamic variables were measured throughout the study: arterial pressure, central venous pressure (CVP), pulse contour-derived cardiac index (CCI), heart rate, and PPV.

Transpulmonary thermodilution measurements were performed at study onset (baseline-1) and after volume expansion using the PiCCO® device. Values were computed as the mean of four consecutive measurements, using a 15-ml bolus of cold saline serum. ΔCCI_TREND_ was computed as the difference between the maximal value of CCI during Trendelenburg and baseline-1 CCI, normalized by baseline-1 CCI. ΔPPV_6-8_ was computed as the difference between the maximal value of PPV during ventilation with VT 8 ml.kg^-1^ and baseline-2 PPV, normalized by baseline-2 PPV. ΔCCI_EEO_ was computed as the difference between the maximal value of CCI during the EEO maneuver and baseline-3 CCI, normalized by baseline-3 CCI. Fluid responsiveness was deemed present if cardiac index assessed by transpulmonary thermodilution (CI_TPTD_) increased by at least 15% after volume expansion, as compared to baseline-1 [20].

### Study endpoints

The study primary endpoint was the diagnostic performance of ΔCCI_TREND_ to predict fluid responsiveness. Secondary endpoints were diagnostic performance of ΔCCI_EEO_ to predict fluid responsiveness, and both PPV and ΔPPV_6-8_.

### Statistical analysis

Statistical analyses were performed using R [21]. Median (1st quartile to 3rd quartile) and counts with percentages are reported for quantitative and categorical variables, respectively. A *p* value below 0.05 was chosen for statistical significance.

We calculated that with a sample size of 33 patients, the study would provide at worst ± 0.15 precision for the 95% confidence interval (CI_95%_) of the area under the receiver operating characteristic (ROC) curve (AUC), assuming a prevalence of fluid responsiveness of 50% [16, 22–24] and an AUC of at least 0.8 [25] (i.e. a lower bound for the AUC CI_95%_ amounting to at least 0.65).

Comparisons between groups of patients were performed with the Fisher’s exact test for categorical variables, and with the *t* test, Mann-Whitney test or analysis of variance (ANOVA) for continuous and ordinal variables when appropriate. Hemodynamic parameters were compared using a linear mixed effects model [26, 27]. Multiple comparisons between experimental conditions and baseline-1 were performed using Dunnett’s test [28].

Diagnostic performance of tests under investigation was assessed by computation of the AUC [29]. The CI_95%_ for the AUC was computed using the Delong method. The optimal cutoffs were computed by maximizing the Youden index. The CIs for optimal cutoffs were computed using the gray zone approach (area of uncertainty of optimal cutoffs) [23]. Response to each test below the lower or above the higher border of the gray zone were considered negative and positive, respectively. Responses to the test within the gray zone were considered inconclusive. The CI_95%_ for sensitivity, specificity and medians were computed using bootstrapping and 10000 replicates [30, 31].

## Results

### Population

During the study period, 55 patients presented with inclusion criteria (see Additional file 3) and 33 were included, whose general characteristics, cardiovascular and respiratory parameters at inclusion are reported in Tables 1 and 2. There were 14 patients (42%) who exhibited cardiac arrhythmia at inclusion and were excluded in the analyses pertaining to PPV: 15 patients (45%) were classified as fluid responsive after fluid administration.

**Table 1:** Patients’ general characteristics at inclusion

Characteristics	Overall population (n = 33)	Fluid non-responders (n = 18)	Fluid Responders (n = 15)	*p*
Age (years)	69 (63–78)	68 (60–71)	74 (66–78)	0.14
Male gender	23 (70%)	12 (67%)	11 (73%)	0.72
SAPS II	58 (49–65)	56 (45–65)	58 (54–62)	0.47
Time between ARDS onset and inclusion (day)	1 (0–3)	1 (0–1)	2 (1–6)	0.03
ARDS category				0.28
• moderate ARDS	10 (30%)	7 (39%)	3 (20%)	
• severe ARDS	23 (70%)	11 (61%)	12 (80%)	
ARDS risk factors^a^				
• pneumonia	23 (72%)	13 (72%)	10 (67%)	1
• non-pulmonary sepsis	5 (15%)	1 (6%)	4 (27%)	0.15
• aspiration of gastric content	5 (15%)	4 (22%)	1 (7%)	0.35
• other	2 (6%)	0 (0%)	2 (13%)	0.20
SOFA score	11 (10–13)	11 (9–13)	12 (10–14)	0.35
Midazolam dose (mg.kg^-1^.h^-1^)	0.10 (0.06–0.13)	0.09 (0.07–0.13)	0.10 (0.06–0.12)	0.90
Morphine dose (mg.kg^-1^.h^-1^)	0.05 (0.04–0.07)	0.04 (0.03–0.07)	0.06 (0.04–0.08)	0.36
ARDS adjunctive therapies				
• NMBA administration	30 (91%)	16 (89%)	14 (93%)	1
• iNO administration	4 (12%)	2 (11%)	2 (13%)	1
• renal replacement therapy	14 (42%)	7 (39%)	7 (47%)	0.73
Clinician justification to administer intravascular fluids^b^				
• arterial lactate >2 mmol.L^-1^	23 (70%)	13 (72%)	10 (67%)	1
• MAP <65 mm Hg	17 (52%)	12 (67%)	5 (33%)	0.08
• cardiac output decrease	12 (36%)	5 (28%)	7 (47%)	0.30
• urine output <0.5 ml.kg.h^-1^	11 (33%)	5 (28%)	6 (40%)	0.49
• heart rate >100 min^-1^	11 (33%)	5 (28%)	6 (40%)	0.49
• skin mottling	10 (30%)	5 (28%)	5 (33%)	1
• 1 of the above criteria	7 (21%)	4 (22%)	3 (20%)	
• > 1 of the above criteria	26 (79%)	14 (78%)	12 (80%)	1
Cause of circulatory failure				
• septic shock	29 (88%)	15 (83%)	14 (93%)	0.61
• cardiogenic shock	2 (6%)	1 (6%)	1 (7%)	1
• other	2 (6%)	2 (11%)	0 (0%)	0.49
Cardiac arrhythmia				1
• atrial fibrillation	7 (21%)	4 (22%)	3 (20%)	
• other	7 (21%)	4 (22%)	3 (20%)	
• none	19 (58%)	10 (56%)	9 (60%)	

Data are median (1st quartile to 3rd quartile) or count (percentage)

*ARDS* acute respiratory distress syndrome, *ICU* intensive care unit, *iNO* inhaled nitric oxide, *MAP* mean arterial pressure, *NMBA* neuromuscular blocking agents, *SAPS II* Simplified Acute Physiology Score II, *SOFA* Sequential Organ Failure Assessment

^a^Total >100% as multiple risk factors could be identified per patient

^b^Total >100% as multiple justifications could be given per patient

**Table 2:** Cardiovascular and respiratory parameters at inclusion

Parameters	Overall population (n = 33)	Fluid non-responders (n = 18)	Fluid responders (n = 15)	*p*
Norepinephrine administration	28 (85%)	17 (94%)	11 (73%)	0.15
Norepinephrine dose (μg.kg^-1^.min^-1^)	0.98 (0.41–1.50)	0.68 (0.35–1.04)	1.32 (0.71–1.97)	0.12
Dobutamine administration	7 (21%)	4 (22%)	3 (20%)	1
Dobutamine dose (μg.kg^-1^.min^-1^)	10.0 (7.5–14.8)	11.4 (8.8–13.8)	10.0 (5.2–14.9)	0.86
Heart rate (min^-1^)	100 (93–115)	102 (92–117)	100 (93–112)	0.69
MAP (mm Hg)	69 (64–72)	67 (62–71)	70 (68–72)	0.14
PPV (%)^a^	7 (5–10)	7 (5–8)	7 (5–11)	0.92
CVP (mm Hg)	7 (5–11)	7 (5–10)	7 (4–11)	0.93
CI_TPTD_ (L.min^-1^.m^-2^)	2.75 (2.06–3.50)	2.94 (2.26–3.50)	2.70 (2.04–3.04)	0.48
GEDVI (ml.m^-2^)	701 (587–854)	697 (586–866)	701 (614–773)	0.89
ELWI (ml.kg^-1^ PBW)	14.6 (11.8–20.4)	15.8 (12.6–20.9)	13.7 (10.1–18.3)	0.23
Global ejection fraction (%)	16 (13–18)	16 (14–20)	16 (13–17)	0.66
Respiratory rate (min^-1^)	30 (28–35)	30 (28–35)	30 (28–35)	0.84
Heart rate/respiratory rate ≤3.6	20 (61%)	10 (56%)	10 (67%)	0.72
Tidal volume (ml.kg^-1^ PBW)	6.0 (5.9–6.1)	6.0 (6.0–6.1)	6.0 (5.9–6.0)	0.31
PEEP (cm H_2_O)	8 (5–10)	8 (6–10)	8 (6–10)	0.67
PEEPt,rs (cm H_2_O)	9 (8–11)	9 (8–11)	10 (8–12)	0.83
Pplat,rs (cm H_2_O)	22 (20–27)	22 (20–28)	22 (18–26)	0.54
Driving pressure (cm H_2_O)	12 (10–15)	13 (10–18)	11 (9–14)	0.23
Cst,rs (mL.cm H_2_O^-1^)	30 (23–39)	28 (21–38)	31 (25–43)	0.26
pH	7.33 (7.27–7.38)	7.31 (7.27–7.36)	7.38 (7.28–7.42)	0.20
PaO_2_/FiO_2_ (Torr)	158 (120–208)	155 (120–167)	207 (109–241)	0.17
PaCO_2_ (Torr)	41 (38–51)	46 (41–52)	38 (36–44)	0.06
Arterial lactate (mmol.L^-1^)	2.5 (1.9–6.4)	2.5 (1.9–6.3)	2.5 (1.8–5.8)	0.94

Data are median (1st quartile to 3rd quartile) or count (percentage)

*CI*_*TPTD*_ cardiac index assessed by transpulmonary thermodilution, *Cst,rs* static compliance of the respiratory system, *CVP* central venous pressure, *ELWI* extravascular lung water index, *FiO*_*2*_ inspired oxygen fraction, *GEDVI* global end-diastolic volume index, *MAP* mean arterial pressure, *PaO*_*2*_ partial pressure of arterial oxygen, *PaCO*_*2*_ partial pressure of arterial carbon dioxide, *PBW* predicted body weight, *PEEP* positive end-expiratory pressure, *PEEPt,rs* total PEEP of the respiratory system, *Pplat,rs* plateau pressure of the respiratory system

^a^For patients without cardiac arrhythmia

### Hemodynamic measurements

The total duration of the study amounted to 26 (24–30) min. None of the variables measured after return to baseline settings (baseline-2 to baseline-4) were significantly different from baseline-1 (Table 3). Mean arterial pressure, CVP, CI_TPTD,_ global end-diastolic volume index and global ejection fraction increased significantly after fluid administration. No adverse event was identified throughout the protocol.

**Table 3:** Hemodynamic parameters in each experimental condition

Variables	Baseline-1	Trendelenburg	Baseline-2	VT 8	Baseline-3	EEO	Baseline-4	VE
HR (min^-1^)	101 (93-115)	100 (93–113)	100 (92–117)	100 (92–114)	101 (92–115)	100 (93–113)	99 (92–115)	98 (90–110)
MAP (mm Hg)	69 (64–72)	-	67 (62–72)	-	66 (62–72)	-	67 (62–73)	78 (71–85)*
PPV (%)^a^	7 (5–10)	-	7 (6–8)	11 (7–16)*	8 (5–9)	-	7 (5–8)	5 (3–10)
CVP (mm Hg)	7 (5–11)	11 (9–15)*	7 (4–11)	7 (4–11)	7 (4–10)	6 (4–10)**	7 (5–11)	9 (7–13)*
CCI (L.min^-1^.m^-2^)	2.8 (2.2–3.4)	3.2 (2.5–3.5)*	2.8 (2.1–3.4)	2.7 (2.1–3.4)	2.6 (2.0–3.4)	2.8 (2.2–3.4)	2.8 (2.0–3.4)	3.1 (2.6–3.7)*
CI_TPTD_ (L.min^-1^.m^-2^)	2.75 (2.06-3.50)	-	-	-	-	-	-	3.16 (2.51–3.84)*
GEDVI (mL.m^-2^)	696 (587–797)	-	-	-	-	-	-	727 (606–894)*
ELWI (mL.kg^-1^ PBW)	14.6 (11.8–20.4)	-	-	-	-	-	-	13.8 (11.7–20.1)
GEF (%)	16 (13–18)	-	-	-	-	-	-	18 (15–22)*

Data are median (1st quartile to 3rd quartile)

*CCI* continuous cardiac index assessed by pulse contour analysis, *CI*_*TPTD*_ cardiac index assessed by transpulmonary thermodilution, *CVP* central venous pressure, *EEO* end-expiratory occlusion, *ELWI* extravascular lung water index, *GEDVI* global end-diastolic volume index, *GEF* global ejection fraction, *HR* heart rate, *MAP* mean arterial pressure, *PBW* predicted body weight, *PPV* pulse pressure variation, *VE* volume expansion, *VT* tidal volume

^a^For patients without cardiac arrhythmia (n = 19)

*p < 0.001 vs. baseline-1; **p < 0.05 vs. baseline-1

### Trendelenburg maneuver

**Fig. 2:**
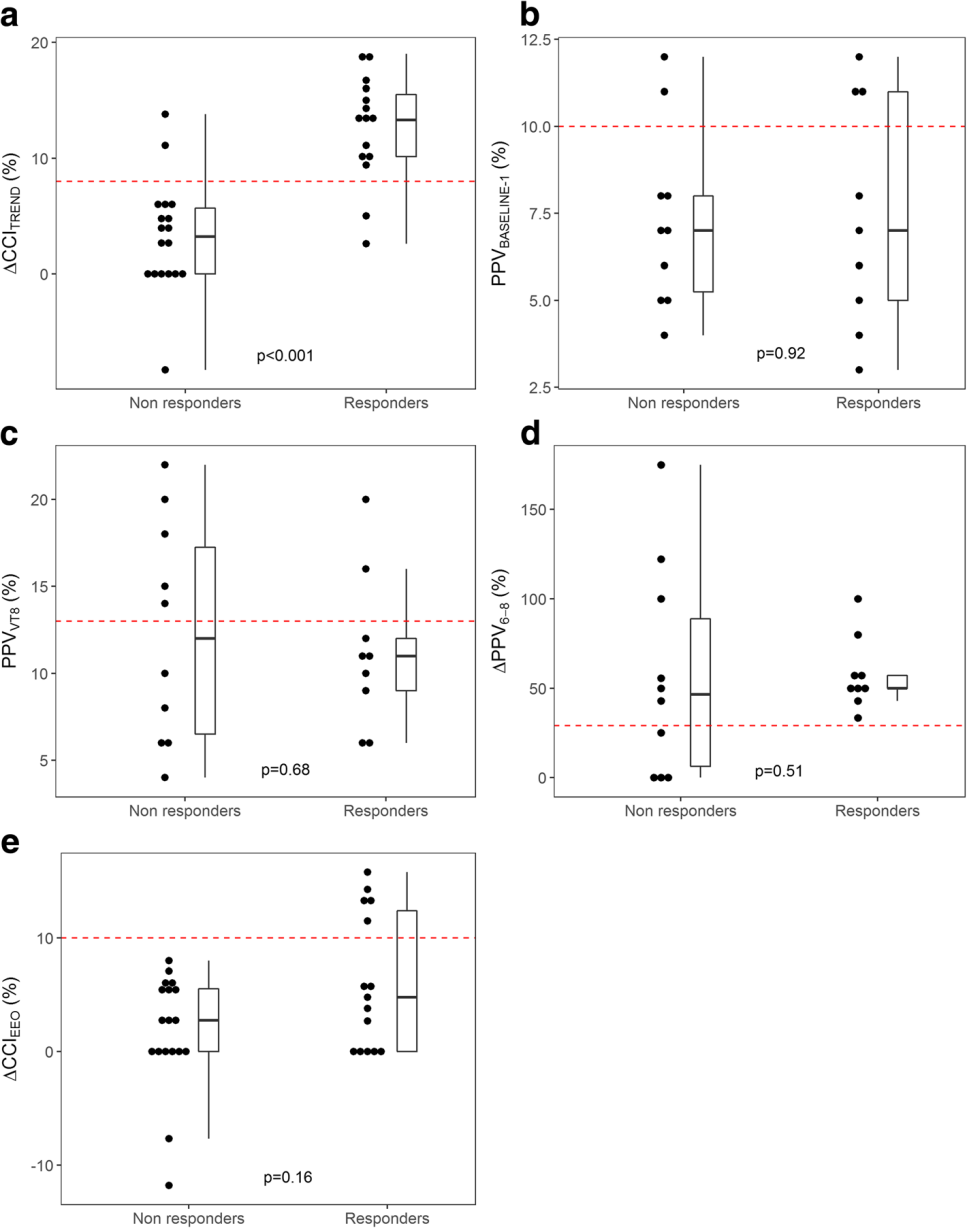
Individual values from five diagnostic tests to detect fluid responsiveness in fluid responders and non-responders. Closed circles are individual values. Red dotted lines display optimal thresholds for each diagnostic test computed by receiver operating characteristic (ROC) curve analysis. **a**. ΔCCI_TREND_, change in continuous cardiac index during the Trendelenburg maneuver; **b**. PPV_BASELINE-1_, pulse pressure variation during ventilation with 6 ml.kg^-1^ predicted body weight tidal volume; **c**. PPV_VT8_, pulse pressure variation during ventilation with 8 ml.kg^-1^ predicted body weight tidal volume; **d**. ΔPPV_6-8_, change in pulse pressure variation between ventilation with 6 and 8 ml.kg^-1^ predicted body weight tidal volume; **e**. ΔCCI_EEO_, change in continuous cardiac index during end-expiratory occlusion

**Fig. 3:**
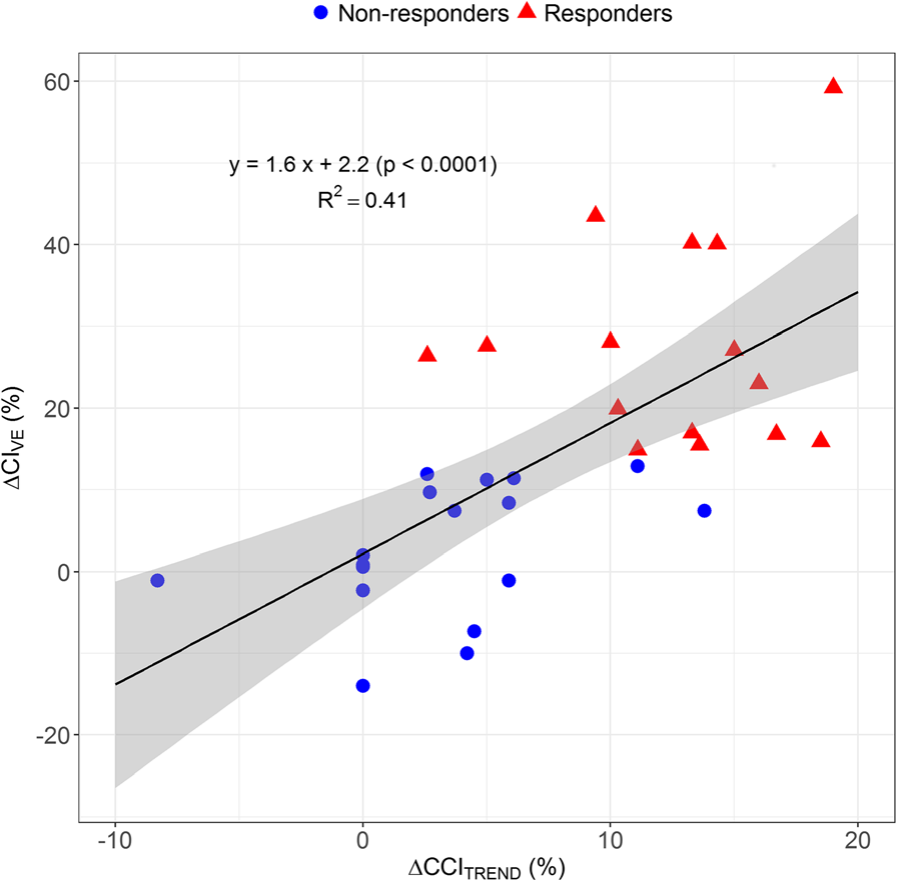
Relationship between change in continuous cardiac index during the Trendelenburg maneuver (ΔCCI_TREND_) and change in transpulmonary thermodilution-cardiac index by volume expansion (ΔCI_VE_). The black line is the regression line. The shaded area is the 95% confidence interval of the regression line. There are 33 data points presented although some data points are overlapping

**Fig. 4:**
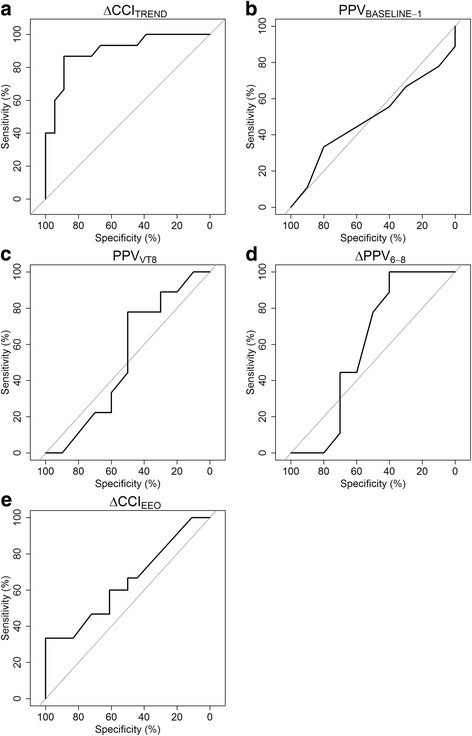
Receiver operating characteristics curves from five diagnostics tests to detect fluid responsiveness. ΔCCI_TREND_, change in continuous cardiac index during the Trendelenburg maneuver; ΔCCI_EEO_, change in continuous cardiac index during end-expiratory occlusion; PPV_BASELINE-1_, pulse pressure variation during ventilation with 6 ml.kg^-1^ predicted body weight tidal volume; PPV_VT8_, pulse pressure variation during ventilation with 8 ml.kg^-1^ predicted body weight tidal volume; ΔPPV_6-8_, change in pulse pressure variation between ventilation with 6 and 8 ml.kg^-1^ predicted body weight tidal volume

**Fig. 5:**
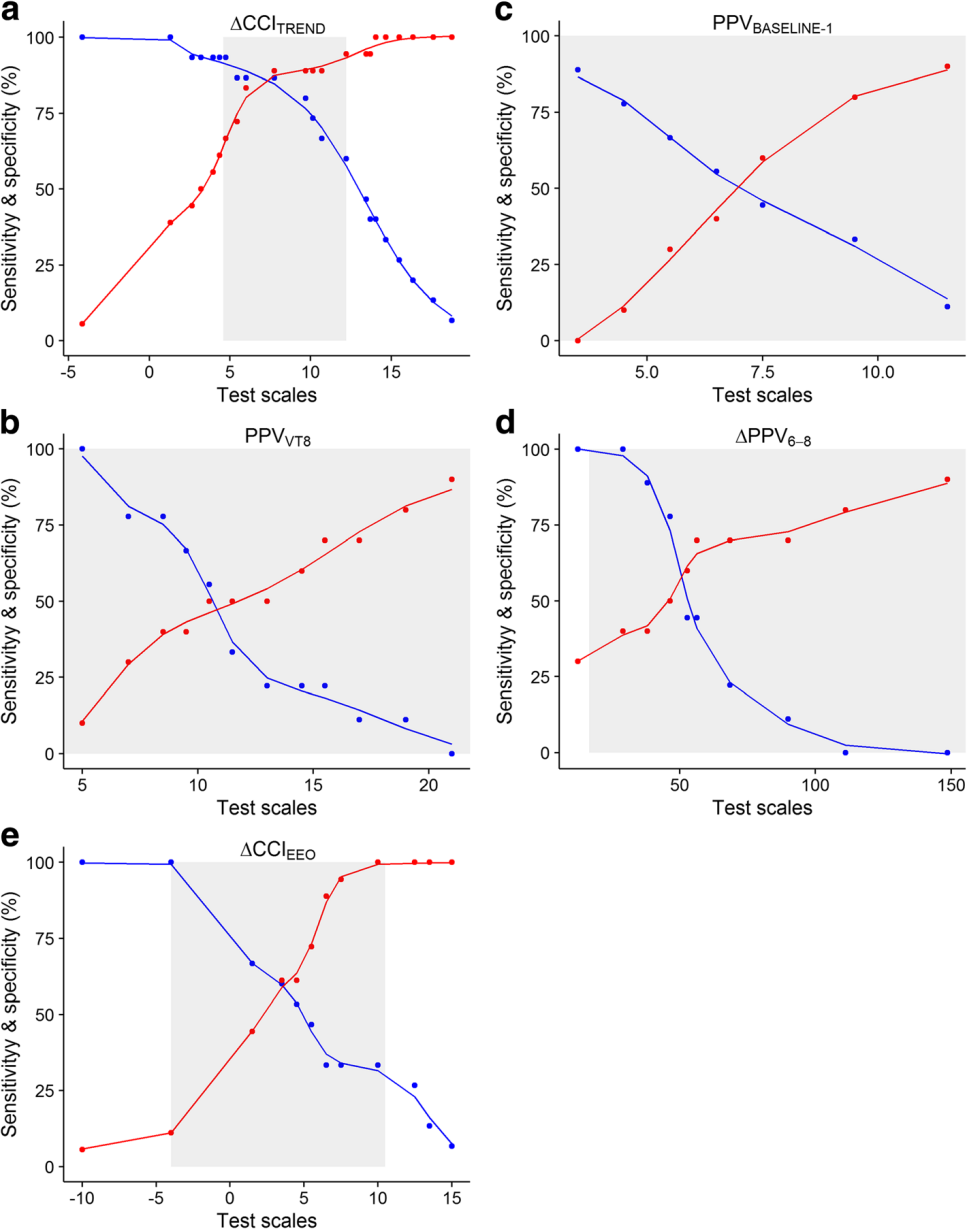
Sensitivity and specificity of each diagnostic test according to the value of the diagnostic cutoff. Blue and red data points are sensitivity and specificity, respectively, computed at each value of the diagnostic cutoff. Blue and red curves are fitted curves to sensitivity and specificity computed values. The 95% confidence intervals for optimal value of cutoff are depicted as a shaded zone (gray zone defining inconclusive response). ΔCCI_TREND_, change in continuous cardiac index during the Trendelenburg maneuver; ΔCCI_EEO_, change in continuous cardiac index during end-expiratory occlusion; PPV_BASELINE-1_, pulse pressure variation during ventilation with 6 ml.kg^-1^ predicted body weight tidal volume; PPV_VT8_, pulse pressure variation during ventilation with 8 ml.kg^-1^ predicted body weight tidal volume; ΔPPV_6-8_, change in pulse pressure variation between ventilation with 6 and 8 ml.kg^-1^ predicted body weight tidal volume

CVP increased significantly during Trendelenburg, while heart rate remained unchanged (Table 3). Median ΔCCI_TREND_ amounted to 6% (CI_95%_, 3–10%) and was significantly greater in responders than in non-responders (13% vs. 3%, *p* < 0.001, Fig. 2). ΔCCI_TREND_ was significantly correlated with change in CI_TPTD_ related to volume expansion (*R* ^2^ = 0.41, Fig. 3). ΔCCI_TREND_ predicted fluid responsiveness with an AUC of 0.90 (CI_95%_, 0.80–1.00, *p* < 0.001), with sensitivity of 87% and specificity of 89% at a threshold of 8% (gray zone, 5–12%) (Table 4, Figs. 4 and 5). Cardiac index response to volume expansion increased stepwise in patients with a negative response, those with an inconclusive response, and those with a positive response to the test (see Additional file 4). Four patients were misclassified (Fig. 2), and none of their hemodynamic and respiratory parameters were significantly different from those of the 29 correctly classified patients (data not shown).

**Table 4:** Diagnostic performance of five diagnostic tests to predict fluid responsiveness

Tests	Number of patients analyzed	AUC (CI_95%_)	Optimal threshold	Gray zone of optimal threshold	Patients in gray zone, (number (%))	Sensitivity (CI_95%_)	Specificity (CI_95%_)	PLR	NLR
ΔCCI_TREND_	33	0.90* (0.80–1.00)	8%	(5–12%)	10 (30%)	87% (67–100%)	89% (72–100%)	7.90	0.15
PPV_BASELINE-1_	19	0.49 (0.21–0.77)	10%	(–Inf to Inf)	19 (100%)	33% (0–67%)	80% (50–100%)	1.65	0.84
PPV_VT8_	19	0.52 (0.24–0.80)	9%	(–Inf to Inf)	19 (100%)	78% (44–100%)	40% (10–70%)	1.30	0.56
ΔPPV_6-8_	19	0.59 (0.31–0.88)	29%	(17%–Inf)	16 (84%)	100% (100–100%)	40% (10–70%)	1.67	0
ΔCCI_EEO_	33	0.65 (0.46–0.84)	10%	(−4% to 11%)	26 (79%)	33% (13–60%)	100 (100–100%)	Inf	0.67

*AUC* area under ROC curve. *CI*_*95%*_ 95% confidence interval, *ΔCCI*_*EE*_ change in continuous cardiac index during end-expiratory occlusion, *ΔCCI*_*TREND*_ change in continuous cardiac index during the Trendelenburg maneuver, *ΔPPV*_*6-8*_ change in pulse pressure variation between ventilation with 6 and 8 ml.kg^-1^ predicted body weight tidal volume, *Inf* infinity, *NLR* negative likelihood ratio, *PLR* positive likelihood ratio, *PPV*_*BASELINE-1*_ pulse pressure variation at baseline-1, *PPV*_*VT8*_ pulse pressure variation during ventilation with 8 ml.kg^-1^ tidal volume

*p < 0.001 vs. an AUC of 0.5

### Pulse pressure variation

PPV_BASELINE-1,_ PPV_VT8_ and ΔPPV_6-8_ did not significantly differ between fluid responders and non-responders (Fig. 2). None of the three PPV-derived diagnostic tests were statistically significant for AUC (Table 4, Fig. 4). ΔPPV_6-8_ exhibited the greatest sensitivity (100% (CI_95%_, 100–100%)) at a threshold of 29%, but with a very low specificity (40% (CI_95%_, 10–70%)).

False positive patients with the PPV_BASELINE-1_ test had significantly greater driving pressure while true negative had significantly lower PaO_2_/FiO_2_ ratio (data not shown). CI_TPTD_ at inclusion was significantly lower in the 5 false positive patients as assessed by PPV_VT8_.

### End-expiratory occlusion

CVP decreased slightly but significantly from 7 (5–11) to 6 (4–10) mm Hg during EEO, while CCI remained unchanged (Table 3). ΔCCI_EEO_ was not significantly different in responders and non-responders (Fig. 2). The AUC of ΔCCI_EEO_ to predict fluid responsiveness amounted to 0.65 (CI_95%_, 0.46–0.84), and was not significantly different from 0.5 (Table 4, Fig. 4). ΔCCI_EEO_ had sensitivity of 33% (CI_95%_, 13–60%) and specificity of 100% (CI_95%_, 100–100%) at a threshold of 10% (gray zone, –4% to 11%) to predict fluid responsiveness (Table 4, Fig. 5).

In the 14 patients with change in CVP (ΔCVP) ≥0 mm Hg, the AUC of ΔCCI_EEO_ amounted to 0.89 (CI_95%_, 0.70–1.00) (*p* < 0.05), while it was not statistically different from 0.5 in the 19 patients with ΔCVP <0 mm Hg (see Additional files 5 and 6).

## Discussion

This study is the first to evaluate the diagnostic performance of several diagnostic tests to predict fluid responsiveness in patients with ARDS in the PP under protective ventilation. The main findings are that: (1) change in cardiac index during the Trendelenburg maneuver is a highly reliable test to predict fluid responsiveness, with both sensitivity and specificity approximating 90%; (2) change in PPV during a transient increase in VT from 6 to 8 ml.kg^-1^ is highly sensitive to predict fluid responsiveness, but with low specificity; and (3) change in cardiac index during EEO has low sensitivity, but high specificity to predict fluid responsiveness in this clinical setting.

### Reliability of the Trendelenburg maneuver to predict fluid responsiveness

In the present study, ΔCCI_TREND_ amounted to 6% (CI_95%_, 3–13%), and was in the range of the 9% increase observed in a recent systematic review [32], although performed in subjects in the supine position, with various degrees of head-down tilt angulation. An important issue in the reliability of the Trendelenburg maneuver to predict fluid responsiveness is related to baroreflex activation in this position, leading to systemic vasodilation, decreased heart rate and myocardial contractility. However, we did not observe a significant change in heart rate in the Trendelenburg position as compared to baseline, in keeping with the results of the aforementioned systematic review [32]. While baroreflex activation is immediately evident after carotid declamping in patients who were awake and undergoing carotid surgery, the maximal effect on heart rate is observed 10 min later [33]. Opposite to this, in healthy volunteers, midazolam has been shown to dose-dependently blunt the fast parasympathetic efferent pathway of the baroreflex [34, 35]. Taken together, these data suggest that the 1-min duration of the maneuver may not have been long enough to significantly activate the baroreflex in deeply sedated patients with ARDS.

### Reliability of PPV to predict fluid responsiveness

The present study confirmed the lack of predictive ability of PPV to predict fluid responsiveness in patients with ARDS under protective ventilation [11–13]. This finding is not unexpected since cardiopulmonary interactions under mechanical ventilation (the underlying physiological mechanism behind PPV) are dependent on both ventilatory settings and transmission of airway pressure to cardiac filling pressures. This transmission is inversely related to respiratory system elastance [15], and linearly related to the ratio of chest wall to respiratory system elastances [36]. In conditions combining low VT and low respiratory system elastance as observed in patients with ARDS under protective ventilation, a high rate of false negative patients is expected.

Performing a VT challenge did not significantly enhance the reliability of the PPV test, since sensitivity increased at the expense of specificity. While this VT challenge increased the reliability of the PPV test in one study performed on 22 ICU patients in the supine position (9% with ARDS) [13], our data suggest that this finding should not be extrapolated to patients with ARDS on PP. Previous studies have shown that false positive patients for PPV may occur in the context of right ventricular failure [7, 37], and the higher driving pressure in this group of the present study favors this hypothesis [38].

### Reliability of EEO to predict fluid responsiveness

The EEO test has been shown to accurately predict fluid responsiveness in the supine position in four studies [12, 14, 39, 40] including one restricted to patients with ARDS ventilated with VT slightly greater than 6 ml.kg^-1^ [14]. However, its predictive performance was poor in a recent study in which 6 ml.kg^-1^ VT was first applied, but was restored during a VT challenge at 8 ml.kg^-1^ [13]. Our results are in line with this study, and the high rate of false negative patients in our study (30%) suggests that the decrease in the cyclic stress applied to the cardiovascular system during ARDS under protective ventilation (due to both low VT and decreased respiratory system compliance) is not sufficient to generate a detectable effect on cardiac index in some patients. We therefore hypothesized that the PP-induced increase in intra-abdominal pressure [41] could generate an impediment to venous return, promoting a zone-2 condition in the inferior vena cava in some patients [42] (and hence a pressure gradient between the inferior vena cava and the right atrium), and could explain the high false negative rate in our study. The slight decrease in CVP during the EEO in 53% of the patients, combined with the low predictive value of ΔCCI_EEO_ in this subpopulation favors this hypothesis, although the lack of direct measurement of intra-abdominal pressure and venous return precludes any definite conclusion. Finally, it should be emphasized that the EEO remains a highly specific test in patients with ARDS under protective ventilation in PP.

### Limitations

Some limitations of the present study should be acknowledged. First, the monocentric feature of this study questions the generalizability of its results. Second, amplifying the postural change during the Trendelenburg maneuver (beyond 13° and −13°) could have maximized the blood transfer from the lower body parts towards the central circulation and may have further increased the sensitivity of this test. Third, the high number of patients with cardiac arrhythmia (therefore excluded from PPV analyses) makes the study strongly underpowered for all analyses pertaining to this test. Fourth, the lack of blinding precludes control of a potential evaluation bias. Fifth, cardiac output assessed from pulse contour analysis may be inaccurate in relation to change in resistive and elastic characteristics of the vascular system, although its reliability is acceptable during the hour following calibration [43]. Finally, the lack of randomization between the three maneuvers performed to predict fluid responsiveness (namely Trendelenburg maneuver, VT challenge and EEO) could have hampered a reliable evaluation of the latter two tests.

### Clinical implications

Risk minimization is an important issue in fluid administration in patients with severe ARDS, given the potential for harm of unnecessary fluid bolus. Using the Trendelenburg test, 70% of the patients could be classified outside the gray zone, meaning that fluid responsiveness was assessed with near certainty in the majority of the patients. Regarding the 30% of patients within the gray zone of the Trendelenburg test, fluid administration may be considered, although response to fluid therapy may be less intense in this group. Finally, whether guiding fluid therapy using indices of fluid responsiveness improve ARDS prognosis remains unknown, although it may help to decrease fluid administration in patients with septic shock [44].

## Conclusions

This study suggests that the Trendelenburg maneuver is reliable to predict fluid responsiveness in patients with ARDS under protective ventilation in the prone position. Pulse pressure variation or change in pulse pressure variation from baseline during a tidal volume challenge, and the end-expiratory occlusion test, although reliable in other clinical settings, did not reach acceptable predictive performance for fluid responsiveness.

## Additional files

Additional file 1: Study protocol. Study protocol as it was submitted to ethics committee and French heath regulation authorities. (DOC 330 kb)

Additional file 2: Figure S1. Protocol description. (DOCX 71 kb)

Additional file 3: Figure S2. Study flow chart. (DOCX 397 kb)

Additional file 4: Figure S3. Change in transpulmonary thermodilution-cardiac index by volume expansion (ΔCI_VE_) as function of response to five diagnostic tests. (DOCX 314 kb)

Additional file 5: Table S1. Diagnostic performance of end-expiratory occlusion to predict fluid responsiveness as a function of change in CVP (ΔCVP) during the test as compared to baseline. (DOCX 14 kb)

Additional file 6: Figure S4. Receiver operating characteristics curves of end-expiratory occlusion to predict fluid responsiveness as a function of change in CVP (ΔCVP) during the test as compared to baseline. (DOCX 67 kb)

Additional file 7: Dataset. (XLSX 53 kb)

## Abbreviations

ARDS: Acute respiratory distress syndrome
AUC: Area under the receiver operating characteristic curve
CCI: Pulse contour-derived cardiac index
CI_95%_: 95% Confidence interval
CI_TPTD_: Transpulmonary thermodilution
CVP: Central venous pressure
EEO: End-expiratory occlusion
PBW: Predicted body weight
PP: Prone position
PPV: Pulse pressure variation
ROC: Receiver operating characteristic
VT: Tidal volume
ΔCCI_TREND_: Difference between the maximal value of CCI during Trendelenburg and baseline-1 CCI, Normalized by baseline-1 CCI
ΔPPV_6-8_: Difference between the maximal value of PPV during ventilation with tidal volume 8 ml.kg^-1^ and baseline-2 PPV, normalized by baseline-2 PPV
ΔCCI_EEO_: Difference between the maximal value of CCI during the EEO maneuver and baseline-3 CCI, normalized by baseline-3 CCI
