# Supplementary material for: Change in cardiac output during Trendelenburg maneuver is a reliable predictor of fluid responsiveness in patients with acute respiratory distress syndrome in the prone position under protective ventilation
Source: Crit Care. 2017 Dec 5;21:295. doi: 10.1186/s13054-017-1881-0 (PMC5718075; doi:10.1186/s13054-017-1881-0)
Supplement: Supplementary file 1 — Study protocol. Study protocol as it was submitted to ethics committee and French heath regulation authorities. (DOC 330 kb) [file 13054_2017_1881_MOESM1_ESM.doc]

**EVALUATION DE LA PERFORMANCE DIAGNOSTIQUE DE PARAMETRES HEMODYNAMIQUES POUR EVALUER LA PRECHARGE-DEPENDANCE AU COURS DU SDRA VENTILE EN DECUBITUS VENTRAL**

***Protocole de Recherche Biomédicale Hors Produit de Santé***

**Promoteur** **:** Hospices Civils de Lyon

BP 2251

3 quai des Célestins,

69229 LYON cedex 02

**Investigateur principal :** RICHARD Jean-Christophe, PU-PH

Service de Réanimation Médicale

Hôpital de la Croix Rousse

103 Grande rue de la Croix Rousse

69004 Lyon

Téléphone : 04 72 07 17 62. Fax : 04 72 07 17 74.

**Investigateurs associés :**

•

• GUERIN Claude, PUPH, chef de service

•

•

• YONIS Hodane, CCA

- TAPONNIER Romain, assistant spécialiste
- PERINEL Sophie, CCA
- AUBLANC Mylène, CCA

Service de Réanimation Médicale

Hôpital de la Croix Rousse

103 Grande rue de la Croix Rousse

69004 Lyon

Téléphone : 04 72 07 17 62. Fax : 04 72 07 17 74.

***Code promoteur : 2012.783***

***Numéro d’enregistrement clinicaltrials.gov : NCTXXXXX***

***Avis favorable du CPP sud-est IV le : 09/07/2013***

***Autorisation de l’ANSM le : 25/06/2013***

RESUME

| **Titre** | Evaluation de la performance diagnostique de paramètres hémodynamiques pour évaluer la précharge-dépendance au cours du SDRA ventilé en décubitus ventral. |
| --- | --- |
| **Promoteur** | Hospices Civils de Lyon  BP 2251  3 quai des Célestins,  69229 LYON cedex 02 |
| **Investigateur principal** | RICHARD Jean-Christophe, PU-PH  Service de Réanimation Médicale  Hôpital de la Croix Rousse  103 Grande rue de la Croix Rousse  69004 Lyon  Téléphone : 04 72 07 17 62. Fax : 04 72 07 17 74. |
| **Version du protocole** | 4 du 21/09/2015 |
| **Justification / Contexte** | Le remplissage vasculaire est un traitement couramment utilisé en réanimation en cas d’altération hémodynamique, avec pour objectif principal d’augmenter le débit cardiaque. L’approche traditionnelle du remplissage vasculaire consiste en la réalisation d’une épreuve de remplissage, qui permet *a posteriori* de détecter une augmentation du débit cardiaque chez 50% des patients, les autres étant exposés à la iatrogénie potentielle du remplissage vasculaire (notamment respiratoire par le biais de la majoration d’un œdème pulmonaire) sans en avoir les bénéfices. Plusieurs épreuves susceptibles de détecter *a priori* la dépendance du débit cardiaque à la précharge ont récemment été développés comme la mesure de l’index cardiaque pendant une épreuve de lever de jambe passif ou une pause télé-expiratoire, ou encore la variation de la pression pulsée. Un ventricule précharge-dépendant étant un ventricule dont le débit cardiaque augmente avec la précharge, et donc augmente avec le remplissage vasculaire. Le problème de ces nouvelles approches est qu’elles sont soit impossibles à réaliser en décubitus ventral, soit non validées dans cette position. Or, l’efficacité du décubitus ventral pour diminuer la mortalité des patients avec SDRA sévère a été suggérée par plusieurs méta-analyses, et récemment confirmée par une étude randomisée multicentrique française initiée par notre équipe. Il est donc probable que le décubitus ventral devienne le « standard of care » de la prise en charge du SDRA sévère dans les années à venir. Il est ainsi absolument nécessaire de développer des méthodes fiables d’évaluation de la précharge-dépendance dans cette position, pour rationaliser le remplissage vasculaire chez les patients avec SDRA, particulièrement exposés à la iatrogénie d’un remplissage vasculaire excessif. Parmi les techniques dérivées de celles utilisées en décubitus dorsal, la mise en position de Trendelenburg en décubitus ventral est le pendant logique de l’épreuve de lever de jambe passif en décubitus dorsal, et mérite d’être évaluée. |
| **Objectif principal** | L’objectif principal de cette étude est d’évaluer la fiabilité de la variation d’index cardiaque induite par la position de Trendelenburg pour prédire la réponse au remplissage vasculaire chez les patients ventilés en décubitus ventral avec SDRA. |
| **Objectifs secondaires** | Les objectifs secondaires de l’étude sont :  - de comparer la fiabilité de 4 méthodes d’évaluation de la précharge-dépendance en décubitus ventral : la variabilité de la pression pulsée à l’état de base, la variabilité de la pression pulsée lors d’une augmentation transitoire du volume courant, la variation d’index cardiaque lors de l’épreuve de Trendelenburg et la variation d’index cardiaque lors d’une occlusion télé-expiratoire  - d’évaluer la tolérance de la position de Trendelenburg en décubitus ventral. |
| **Méthodologie / Schéma de la recherche** | Il s'agit d'une étude monocentrique, exploratoire, d'évaluation de la performance diagnostique de paramètres hémodynamiques au cours du SDRA. |
| **Critères de jugement principal** | Le critère de jugement principal sera la performance diagnostique de la variation d’index cardiaque pendant le passage en position de Trendelenburg (par rapport à l’index cardiaque de base), appréciée par l’aire sous la courbe ROC et son intervalle de confiance à 95%. |
| **Critères de jugement secondaires** | Deux critères de jugement secondaires seront évalués : - la comparaison des aires sous la courbe ROC de 4 paramètres d’évaluation de la précharge dépendance : variabilité de la pression pulsée à l’état de base, variabilité de la pression pulsée après augmentation du volume courant, variation de l’index cardiaque pendant l’occlusion télé-expiratoire, variation de l’index cardiaque pendant la mise en Trendelenburg.  - la tolérance de la position de Trendelenburg |
| **Population cible** | Patients présentant un SDRA selon la définition de Berlin ^1^: 1- insuffisance respiratoire aiguë dans la semaine suivant une agression pulmonaire, 2- opacités pulmonaires bilatérales non expliquées par des épanchements pleuraux, des atélectasies ou des nodules, 3- hypoxémie avec PaO2/FiO2 < 300 sous PEEP ≥ 5 cm d’H2O, 4- non expliquée par une insuffisance cardiaque ou une hypervolémie. |
| **Critères d’inclusion** | - Patient sous ventilation mécanique, en décubitus ventral, avec SDRA - Indication de remplissage vasculaire posée par le clinicien en charge du patient  - Patient en décubitus ventral sous ventilation mécanique en ventilation assistée contrôlée  - Absence d’effort inspiratoire détectable lors des occlusions télé-expiratoires et télé-inspiratoires de 3 secondes  - Cathéter veineux central implanté en territoire cave supérieur fonctionnel  - Cathéter artériel de type Picco implanté en position fémorale  - Patient affilié à un régime de sécurité sociale  - Consentement signé par un proche |
| **Critères de non inclusion** | - Age inférieur à 18 ans  - Contre-indication à la mise en position de Trendelenburg (hypertension intracrânienne documentée ou pathologie neurologique à risque d’hypertension intracrânienne)  - Obstruction cave inférieure  - Patient précédemment inclus dans le même protocole de recherche  - Patient sous mesure de protection administrative et judiciaire |
| **Critères de sortie d’étude** | Le patient sort de l’étude en cas de survenue d’un événement indésirable grave en cours de procédure, ou en cas de retrait de consentement. |
| **Procédures** | L’étude comporte 8 temps expérimentaux de mesures hémodynamiques et respiratoires : à l’état de base en décubitus ventral (**BASE 1**), lors d’une augmentation transitoire du volume courant pendant 1 minute (**VT8**), après le retour en position de repos (**BASE 2**), en position de Trendelenburg pendant 1 minute (**TREND**), après le retour en position de repos (**BASE** 3), à la fin d’une occlusion télé-expiratoire de 15 secondes (**OCC**), après le retour en position de repos (**BASE** 4), et une mesure finale après remplissage vasculaire par 500 ml de Ringer lactate en 20 minutes (**FIN**).  Les mesures hémodynamiques sont réalisées avec le matériel utilisé pour le soin au patient et consistent en des mesures de pression artérielle, de pression veineuse centrale, d’index cardiaque, du volume télédiastolique global indexé, et de l’eau pulmonaire extravasculaire indexée par thermodilution. Les mesures respiratoires sont des mesures de pression expiratoire positive totale et de pression de plateau télé-inspiratoire, et sont renseignées sur l’écran du respirateur en en pressant les boutons appropriés pour réaliser des pauses télé-inspiratoire et télé-expiratoire. |
| **Rapport bénéfices/risques** | Absence de bénéfice attendu à la participation dans l’étude. La seule procédure à risque liée au protocole est la position de Trendelenburg, dont le risque est de majorer une hypertension intracrânienne, qui est un critère d’exclusion. |
| **Nombre de patients** | 33 |
| **Faisabilité** | La faisabilité de l’étude est importante dans la mesure où 30-35 patients avec SDRA sévère (donc justifiant du décubitus ventral) sont hospitalisés annuellement dans le service de réanimation médicale et d’assistance respiratoire. La quasi-totalité de ces patients est sédaté, monitorée avec un cathéter artériel de Picco, et donc potentiellement incluables dans l’étude sous réserve de la signature du consentement éclairé. Toutes les techniques utilisées sont parfaitement maitrisées par les co-investigateurs puisque réalisées quotidiennement dans le cadre du soin aux patients. |
| **Durée de l’étude** | Durée de la période d’inclusion : 4 ans  Durée de participation pour chaque patient : 30 minutes environ  Durée totale de l’étude : 4 ans  Début des inclusions : octobre 2013  Une demande de prolongation de 2 ans a été demandée en raison d’un rythme d’inclusion inférieur à la prévision. |
| **Lieu de la recherche** | Service de réanimation médicale et d’assistance respiratoire – Hôpital de la Croix-Rousse. 69007 LYON |
| **Retombées attendues** | Amélioration de la prise en charge des patients avec SDRA, avec rationalisation des décisions de remplissage vasculaire, évitement des remplissages vasculaires inutiles à l’origine d’une iatrogénie notamment respiratoire (majoration de l’œdème pulmonaire) |

**LISTE DES ABREVIATIONS**

| **ANSM** | Agence Nationale de Sécurité du Médicament et des produits de santé |
| --- | --- |
| **ARC** | Attaché de Recherche Clinique |
| **BPC** | Bonnes Pratiques Cliniques |
| **CNIL** | Commission Nationale Informatique et Liberté |
| **CPP** | Comité de Protection des Personnes |
| **CRF** | Case Report Form (cahier d’observation) |
| **EIG** | Effet Indésirable Grave |
| **EIGI** | Effet Indésirable Grave Inattendu |
| **EvIG** | Evènement Indésirable Grave |
| **ICh** | International Conference on Harmonisation |
| **MR** | Méthodologie de Référence |
|  |  |
|  |  |

Table des matières

[1 Informations générales 9](#__RefHeading___Toc360093338)

[1.1 Numéro d’enregistrement de la recherche 9](#__RefHeading___Toc360093339)

[1.2 Titre complet 9](#__RefHeading___Toc360093340)

[1.3 Promoteur 9](#__RefHeading___Toc360093341)

[1.4 Investigateurs 10](#__RefHeading___Toc360093342)

[2 Justification scientifique et description générale de la recherche 11](#__RefHeading___Toc360093343)

[2.1 Résumé des résultats des essais non cliniques et des essais cliniques disponibles et pertinents au regard de la recherche biomédicale concernée 11](#__RefHeading___Toc360093344)

[2.2 Résumé des bénéfices, le cas échéant, et des risques prévisibles et connus pour les personnes se prêtant à la recherche 12](#__RefHeading___Toc360093345)

[2.3 Description de la population à étudier 12](#__RefHeading___Toc360093346)

[2.4 Références à la littérature scientifique et aux données pertinentes servant de référence pour la recherche 13](#__RefHeading___Toc360093347)

[3 Objectifs de la recherche 13](#__RefHeading___Toc360093348)

[4 Conception de la recherche. 13](#__RefHeading___Toc360093349)

[4.1 Énoncé précis des critères d’évaluation principaux et, le cas échéant, des critères d'évaluation secondaires 13](#__RefHeading___Toc360093350)

[4.2 Description de la méthodologie de la recherche, accompagnée de sa présentation schématique précisant notamment les visites et les examens prévus 13](#__RefHeading___Toc360093351)

[4.3 Description des mesures prises pour réduire et éviter les biais incluant notamment les méthodes de mise en insu 13](#__RefHeading___Toc360093352)

[4.4 Procédures 14](#__RefHeading___Toc360093353)

[4.4.1 Temps expérimentaux 14](#__RefHeading___Toc360093354)

[4.4.2 Modalités des mesures hémodynamiques 14](#__RefHeading___Toc360093355)

[4.4.3 Modalités des mesures respiratoires 14](#__RefHeading___Toc360093356)

[4.5 Durée prévue de participation des personnes, et description de la chronologie et de la durée de toutes les périodes de la recherche, y compris le suivi, le cas échéant 14](#__RefHeading___Toc360093357)

[4.6 Description des règles d’arrêt définitif ou temporaire 15](#__RefHeading___Toc360093358)

[4.6.1 a) de la participation d’une personne à la recherche 15](#__RefHeading___Toc360093359)

[4.6.2 b) d’une partie ou de la totalité de la recherche 15](#__RefHeading___Toc360093360)

[4.7 Dispositions mises en œuvre en vue du maintien de l’insu et procédures de levée de l’insu, le cas échéant 15](#__RefHeading___Toc360093361)

[4.8 Identification de toutes les données à recueillir directement dans les cahiers d’observation, qui seront considérées comme des données source. 15](#__RefHeading___Toc360093362)

[5 Sélection et exclusion des personnes de la recherche 17](#__RefHeading___Toc360093363)

[5.1 Critères d’inclusion 17](#__RefHeading___Toc360093364)

[5.2 Critères d'exclusion 18](#__RefHeading___Toc360093365)

[5.3 Procédure d’arrêt prématuré de la recherche ou d’exclusion pour une personne de la recherche et procédure de suivi de la personne 18](#__RefHeading___Toc360093366)

[6 Traitement administré aux personnes qui se prêtent à la recherche 18](#__RefHeading___Toc360093367)

[7 Évaluation de l’efficacité 18](#__RefHeading___Toc360093368)

[7.1 Calendrier prévus pour mesurer, recueillir et analyser les paramètres d'évaluation de l’efficacité. 18](#__RefHeading___Toc360093369)

[8 Évaluation de la sécurité 18](#__RefHeading___Toc360093370)

[8.1 Définitions 18](#__RefHeading___Toc360093371)

[8.1.1 Evénement indésirable 18](#__RefHeading___Toc360093372)

[8.1.2 Evénement indésirable grave (EIG) 18](#__RefHeading___Toc360093373)

[8.2 Modalités de détection et de recueil des événements indésirables 19](#__RefHeading___Toc360093374)

[8.3 Responsabilités de l’investigateur 19](#__RefHeading___Toc360093375)

[8.3.1 Déclaration des EIG 19](#__RefHeading___Toc360093376)

[8.3.2 Evaluation de la causalité 20](#__RefHeading___Toc360093377)

[8.4 Responsabilités du promoteur 20](#__RefHeading___Toc360093378)

[8.5 Constitution d’un comité de surveillance indépendant 20](#__RefHeading___Toc360093379)

[9 Statistiques 20](#__RefHeading___Toc360093380)

[9.1 Description des méthodes statistiques prévues, y compris du calendrier des analyses intermédiaires prévues 20](#__RefHeading___Toc360093381)

[9.1.1 Analyse intermédiaire 20](#__RefHeading___Toc360093382)

[9.1.2 Caractéristiques de la population lors de l’inclusion 20](#__RefHeading___Toc360093383)

[9.1.3 Analyse des critères de jugement 20](#__RefHeading___Toc360093384)

[9.2 Nombre prévu de personnes à inclure dans la recherche, et nombre prévu de personnes dans chaque lieu de recherches avec sa justification statistique 20](#__RefHeading___Toc360093385)

[9.3 Degré de signification statistique prévu 21](#__RefHeading___Toc360093386)

[9.4 Critères statistiques d’arrêt de la recherche 21](#__RefHeading___Toc360093387)

[9.5 Méthode de prise en compte des données manquantes, inutilisées ou non valides 21](#__RefHeading___Toc360093388)

[9.6 Gestion des modifications apportées au plan d’analyse de la stratégie initiale 21](#__RefHeading___Toc360093389)

[9.7 Toute modification apportée par la suite au plan d’analyse statistique devra être justifiée et donnera lieu à une nouvelle version du document. Ces déviations au plan d’analyse seront reportées dans le rapport final de l’étude. L’ensemble des documents sera conservé dans le dossier de l’étude.Choix des personnes à inclure dans les analyses 21](#__RefHeading___Toc360093390)

[10 Droit d'accès aux données et documents source. 21](#__RefHeading___Toc360093391)

[10.1 Accès aux données : 21](#__RefHeading___Toc360093392)

[10.2 - les investigateurs mettront à disposition des personnes chargées du suivi, du contrôle de qualité ou de l'audit de la recherche biomédicale, les documents et données individuelles strictement nécessaires à ce contrôle, conformément aux dispositions législatives et réglementaires en vigueur (articles L.1121-3 et R.5121-13 du code de la santé publique).Documents sources 21](#__RefHeading___Toc360093393)

[10.3 Confidentialité des données 22](#__RefHeading___Toc360093394)

[11 Contrôle et assurance de la qualité 22](#__RefHeading___Toc360093395)

[12 Considérations éthiques. 23](#__RefHeading___Toc360093396)

[12.1 Autorités compétentes 23](#__RefHeading___Toc360093397)

[12.2 Modifications substantielles 23](#__RefHeading___Toc360093398)

[12.3 Information du patient et formulaire de consentement écrit 23](#__RefHeading___Toc360093399)

[12.4 Période d’exclusion 24](#__RefHeading___Toc360093400)

[12.5 Prise en charge relative à la recherche 24](#__RefHeading___Toc360093401)

[12.6 Indemnisation des sujets 24](#__RefHeading___Toc360093402)

[12.7 Inscription au fichier national des personnes se prêtant à une recherche biomédicale 24](#__RefHeading___Toc360093403)

[13 Traitement des données et conservation des documents et des données relatives à la recherche. 24](#__RefHeading___Toc360093404)

[13.1 Cahier d’observation 24](#__RefHeading___Toc360093405)

[13.2 Saisie et analyse des données 24](#__RefHeading___Toc360093406)

[13.3 CNIL 24](#__RefHeading___Toc360093407)

[13.4 Archivage 24](#__RefHeading___Toc360093408)

[14 Financement et assurance 25](#__RefHeading___Toc360093409)

[14.1 Financement 25](#__RefHeading___Toc360093410)

[14.2 Assurance 25](#__RefHeading___Toc360093411)

[15 Règles relatives à la publication. 25](#__RefHeading___Toc360093412)

[16 Calendrier prévisionnel 25](#__RefHeading___Toc360093413)

[17 Bibliographie 25](#__RefHeading___Toc360093414)

# Informations générales

## Numéro d’enregistrement de la recherche

Identifiants du projet et historique des mises à jour :

Code promoteur : 2012.783

Numéro d’enregistrement clinicaltrials.gov : NCTXXXXX

Avis favorable du CPP sud-est IV le : 09/07/2013

Autorisation de l’ANSM le : 25/06/2013 sous le numéro : IDRCB2013-A00526-39

| **Version** | **Date** | **Motif de mise à jour** |
| --- | --- | --- |
| Version 2 | 27/06/2013 | Remarques CPP du 29 mai 2013 |
| Version 3 | 06/11/2014 | Prolongation étude |
| Version 4 | 21/11/2014 | Prolongation étude |

## Titre complet

Evaluation de la performance diagnostique de paramètres hémodynamiques pour évaluer la précharge-dépendance au cours du SDRA ventilé en décubitus ventral

**Titre abrégé** : PRD-SDRA-DV

**Numéro de code attribué par le promoteur** : 2012.783

**Numéro de version du protocole** : Version 4

**Date du protocole** : 6 novembre 2014

## Promoteur

- *Identité :*

Hospices Civils de Lyon

BP 2251

3 Quai des Célestins

69229 LYON Cedex 02

- *Signature du protocole au nom du Promoteur :*

Muriel MALBEZIN, Directeur de la Recherche Clinique

Hospices Civils de Lyon, Délégation à la Recherche Clinique et à l’Innovation, Siège Administratif, BP 2251, 3 Quai des Célestins, 69229 LYON Cedex 02

Tél : 04 72 40 68 52, Fax : 04 72 40 68 69

- *Responsable de la recherche au niveau du Promoteur :*

Valérie PLATTNER, médecin référent

Hospices Civils de Lyon, Délégation à la Recherche Clinique et à l’Innovation, Siège Administratif, BP 2251, 3 Quai des Célestins, 69229 LYON Cedex 02

Tél : 04 72 40 68 40, Fax : 04 72 11 51 90

- *Responsable de la vigilance des essais au niveau du Promoteur :*

Delphine BERTRAM, pharmacien référent

Hospices Civils de Lyon, Délégation à la Recherche Clinique et à l’Innovation, Siège Administratif, BP 2251, 3 Quai des Célestins, 69229 LYON Cedex 02

Tél : 04 72 40 68 26, Fax : 04 72 11 51 90

## Investigateurs

Il s’agit d’une étude monocentrique, réalisée par le service de Réanimation Médicale, Hôpital de la Croix Rousse, 103 Grande rue de la Croix Rousse, 69004 Lyon. Téléphone : 04 72 07 17 62. Fax : 04 72 07 17 74.

Investigateur principal : Pr Jean-Christophe RICHARD.

Les co-investigateurs sont les suivants :

• GUERIN Claude, PUPH, chef de service

•

• YONIS Hodane, CCA

- TAPONNIER Romain, assistant spécialiste
- PERINEL Sophie, CCA
- AUBLANC Mylène, CCA

# Justification scientifique et description générale de la recherche

## Résumé des résultats des essais non cliniques et des essais cliniques disponibles et pertinents au regard de la recherche biomédicale concernée

Le remplissage vasculaire est un traitement couramment utilisé en réanimation en cas d’altération hémodynamique, avec pour objectif principal d’augmenter le débit cardiaque. L’approche traditionnelle du remplissage vasculaire consistait en la réalisation d’une épreuve de remplissage, dont l’efficacité était jugée à posteriori (augmentation du débit cardiaque) ^2^. Cette approche a été remise en cause dans la mesure où elle n’est associée d’un bénéfice que chez 50% des patients ^3^, le restant subissant le remplissage sans en avoir les bénéfices. Il est même probable que des remplissages vasculaires inadéquats puissent aggraver le pronostic des patients de réanimation. Ainsi, une balance hydrique positive au cours du choc septique est un facteur de risque indépendant de mortalité ^4^. Chez l’enfant, un essai randomisé récent dans le contexte du sepsis sévère a objectivé une surmortalité dans le bras de l’étude où un remplissage vasculaire abondant était attribué ^5^. Finalement, une balance hydrique positive est associée à une augmentation significative de la durée de ventilation mécanique au cours de l’acute lung injury et du syndrome de détresse respiratoire aiguë (SDRA) ^6,7^. A l’inverse, la non réalisation d’un remplissage vasculaire chez les malades susceptibles d’augmenter leur débit cardiaque est préjudiciable, et peut aboutir à l’apparition d’une défaillance multiviscérale. Il y a donc un besoin d’utiliser des paramètres fiables permettant de prédire l’efficacité du remplissage vasculaire ^8^.

La prédiction de l’efficacité du remplissage vasculaire en réanimation a d’abord été appréhendée par l’évaluation indirecte de la volémie (la mesure directe étant impossible), en utilisant la mesure des volumes des cavités cardiaques, ou des pressions de remplissage ventriculaires droite ou gauche. Ces paramètres se sont avérés de faible fiabilité, et ont été remplacés par des paramètres susceptibles de détecter la dépendance à la précharge ^9^. Un ventricule précharge-dépendant étant un ventricule dont le débit cardiaque augmente avec la précharge, et donc augmente avec le remplissage vasculaire ^8^. La mesure de la variabilité de la pression pulsée ^10^ est un paramètre fiable pour évaluer la précharge-dépendance, et donc pour prédire à priori l’efficacité d’une épreuve de remplissage vasculaire. Une méta-analyse de 14 études ^3^ à récemment montré que les sensibilité et spécificité de la variabilité de la pression pulsée pour prédire la réponse au remplissage vasculaire étaient respectivement de 89 et 88%. Le principal écueil dans l’utilisation de ce paramètre est sa moindre fiabilité lors de l’utilisation d’une ventilation mécanique avec des volumes courant inférieurs à 8 ml/kg de poids prédit par la taille ^11,12^, qui sont pourtant recommandés pour ventiler les patients avec SDRA ^13,14^. Une augmentation transitoire du volume courant pour évaluer la variabilité de la pression pulsée a été proposée, mais n’a jamais été formellement validée.

Dans les conditions de ventilation à petit volume courant, il a été proposé de mesurer les effets sur le débit cardiaque d’une épreuve de lever de jambe passif ^15,16^, qui réalise une épreuve de remplissage « interne » en mobilisant le volume sanguin contenu dans les membres inférieurs. Lorsque le débit cardiaque est mesuré en continu lors de cette épreuve, une méta-analyse récente de 8 études a permis d’estimer une sensibilité de 89% et une spécificité de 91% pour prédire la réponse au remplissage vasculaire ^17^. Le problème de cette épreuve de lever de jambe passif est qu’elle est impossible à réaliser en décubitus ventral. Or, l’efficacité du décubitus ventral pour diminuer la mortalité des patients avec SDRA sévère a été suggérée par plusieurs méta-analyses ^18–24^, et récemment confirmée par une étude randomisée multicentrique française récemment publiée, et initiée par notre équipe **^25^**. Il est donc probable que le décubitus ventral devienne le « standard of care » de la prise en charge du SDRA sévère dans les années à venir. Il est ainsi absolument nécessaire de développer des méthodes fiables d’évaluation de la précharge-dépendance dans cette position, pour rationaliser le remplissage vasculaire chez les patients avec SDRA. La mise en position de Trendelenburg en décubitus ventral est le pendant logique de l’épreuve de lever de jambe passif en décubitus dorsal, et mérite d’être évaluée. La mesure de l’index cardiaque lors d’une occlusion télé-expiratoire ^26^ est aussi une option possible pour évaluer la précharge-dépendance en décubitus ventral, mais n’a été évaluée à ce jour qu’en décubitus dorsal.

L’objectif principal de cette étude est donc d’évaluer la fiabilité de la variation d’index cardiaque induite par la position de Trendelenburg pour prédire la réponse au remplissage vasculaire chez les patients ventilés en décubitus ventral avec SDRA.

L’objectif secondaire de l’étude est de comparer la fiabilité de 4 méthodes d’évaluation de la précharge-dépendance en décubitus ventral : la variabilité de la pression pulsée à l’état de base, la variabilité de la pression pulsée lors d’une augmentation transitoire du volume courant, la variation d’index cardiaque lors de l’épreuve de Trendelenburg et la variation d’index cardiaque lors d’une occlusion télé-expiratoire.

## Résumé des bénéfices, le cas échéant, et des risques prévisibles et connus pour les personnes se prêtant à la recherche

Il s'agit d'une étude physiologique de courte durée visant à évaluer la performance diagnostique de paramètres hémodynamiques visant à prédire l’efficacité du remplissage vasculaire au cours du SDRA. Il n'y a aucun bénéfice à attendre de l'inclusion dans le protocole. Pour la même raison, il n’y a aucun risque encouru par les personnes se prêtant à la recherche. Toutes les mesures réalisées sont faites couramment dans le cadre du soin au patient. Les pauses télé-inspiratoires et télé-expiratoires requises pendant certaines mesures font parties du monitoring de la ventilation artificielle et doivent être mesurées de façon répétitive chez tous les malades ventilés avec SDRA pour s’assurer de la sécurité des paramètres ventilatoires utilisés ^14^. L’augmentation transitoire du volume courant à 8 ml/kg requise pendant un temps expérimental reste dans les limites de ce qui est recommandé pour la ventilation des patients avec SDRA ^14^. La mise en position de Trendelenburg n’est potentiellement délétère que chez les patients qui ont une hypertension intracrânienne qui seront exclus de l’étude, et est actuellement proposée chez les patients de réanimation qui présentent une hypotension, dans l’attente de l’efficacité d’un remplissage vasculaire. De plus, la réalisation d’un changement postural pour évaluer l’hémodynamique est maintenant couramment utilisée dans le cadre du soin chez le patient en décubitus dorsal. Enfin, le remplissage vasculaire reçu par le malade n’est pas lié à l’inclusion dans l’étude, mais voit son indication posée par le clinicien en charge du malade.

Au total, la balance bénéfices/risques n’apparait pas défavorable.

## Description de la population à étudier

La population étudiée est constituée de patients avec SDRA selon la définition de Berlin ^1^ : 1- insuffisance respiratoire aiguë dans la semaine suivant une agression pulmonaire, 2- opacités pulmonaires bilatérales non expliquées par des épanchements pleuraux, des atélectasies ou des nodules, 3- hypoxémie avec PaO2/FiO2 < 300 sous PEEP ≥ 5 cm d’H2O, 4- non expliquée par une insuffisance cardiaque ou une hypervolémie.

L'inclusion dans le protocole sera restreinte aux patients ventilés en décubitus ventral, présentant une indication de remplissage vasculaire posée par le clinicien en charge du patient (cf. critères d’inclusion).

## Références à la littérature scientifique et aux données pertinentes servant de référence pour la recherche

cf. chapitre bibliographie en fin de manuscrit.

# Objectifs de la recherche

L'objectif principal de l'étude est d’évaluer la performance diagnostique de paramètres hémodynamiques visant à prédire l’efficacité du remplissage vasculaire pour améliorer l’hémodynamique des patients.

L'hypothèse de travail est que le passage en position de Trendelenburg permet de détecter de façon fiable les patients qui vont ultérieurement bénéficier du remplissage vasculaire (c'est-à-dire augmenter leur index cardiaque après remplissage vasculaire).

Les objectifs secondaires de l'étude sont :

- d’évaluer la performance diagnostique relative de la variation de l’index cardiaque pendant la mise en Trendelenburg et de trois autres tests dont l’utilisation est validée en décubitus dorsal (variabilité de la pression pulsée à l’état de base, variabilité de la pression pulsée après augmentation du volume courant, variation du débit cardiaque pendant une occlusion télé-expiratoire).

- d’évaluer la tolérance de la position de Trendelenburg en décubitus ventral.

# Conception de la recherche.

Il s'agit d'une étude monocentrique, exploratoire, d'évaluation de la performance diagnostique de paramètres hémodynamiques au cours du SDRA.

## Énoncé précis des critères d’évaluation principaux et, le cas échéant, des critères d'évaluation secondaires

Le critère de jugement principal sera la performance diagnostique de la variation d’index cardiaque pendant le passage en position de Trendelenburg (par rapport à l’index cardiaque de base), appréciée par l’aire sous la courbe ROC et son intervalle de confiance à 95%.

Deux critères de jugement secondaires seront évalués :

- la comparaison des aires sous la courbe ROC de 4 paramètres d’évaluation de la précharge dépendance : variabilité de la pression pulsée à l’état de base, variabilité de la pression pulsée après augmentation du volume courant, variation de l’index cardiaque pendant l’occlusion télé-expiratoire, variation de l’index cardiaque pendant la mise en Trendelenburg.
- la tolérance de la position de Trendelenburg

## Description de la méthodologie de la recherche, accompagnée de sa présentation schématique précisant notamment les visites et les examens prévus

L'étude est construite selon un plan expérimental à mesures répétées, sous la forme de la mesure de 4 paramètres avant remplissage vasculaire, et de l’évaluation finale après remplissage vasculaire

## Description des mesures prises pour réduire et éviter les biais incluant notamment des méthodes de mise en insu

La mise en insu est impossible lors de l'acquisition des données. La mesure des 4 paramètres dont la performance diagnostique est étudiée se fait avant la connaissance de l’efficacité du remplissage vasculaire, ce qui minimise les risques de biais lors de leur mesure.

## Procédures

### Temps expérimentaux

L’étude comporte 8 temps expérimentaux de mesures hémodynamiques et respiratoires :

- Mesures à l’état de base en décubitus ventral proclive à 13 ° (**BASE 1**). Le degré d’inclinaison est renseigné par un capteur intégré au lit du malade.
- Mesures lors d’une augmentation transitoire du volume courant à 8 ml/kg de poids prédit par la taille pendant 1 minute (**VT8**)
- Mesure 2 minutes après le retour en position de repos (**BASE 2**)
- Mesure en position de Trendelenburg pendant 1 minute à -13°(**TREND**). Le degré d’inclinaison est renseigné par un capteur intégré au lit du malade.
- Mesure 2 minutes après le retour en position de repos (**BASE** 3)
- Mesure à la fin d’une occlusion télé-expiratoire de 15 secondes (**OCC**)
- Mesure 2 minutes après le retour en position de repos (**BASE** 4)
- Mesure finale après remplissage vasculaire par 500 ml de Ringer lactate en 20 minutes (**FIN**)

### Modalités des mesures hémodynamiques

Les mesures de pression artérielle et de pression veineuse centrale sont réalisées avec le matériel utilisé pour le soin au patient, en plaçant les capteurs à la surface du lit du malade pour éviter les biais de mesure liés au changement posturaux. La qualité des signaux sera évaluée selon la méthode habituelle (calibration à 0 mm de Hg, évaluation de l’absence de sur ou de sous-amortissement lors d’un flush de sérum physiologique). Les mesures seront réalisées pendant la période télé-expiratoire, comme habituellement. La mesure de ces paramètres fait partie du monitoring habituel des patients de réanimation.

La mesure de l’index cardiaque par thermodilution, du volume télédiastolique global indexé, et de l’eau pulmonaire extravasculaire indexée se fait en utilisant le système PICCO, après une calibration par 4 aliquots de 15 ml de sérum physiologique à 4°C. Cette calibration est réalisée toutes les 4 heures dans notre unité, pour conserver la fiabilité de la mesure continue de l’index cardiaque, et sera réalisé juste avant la première condition (Base 1), et juste avant la dernière condition (Fin). La mesure de l’index cardiaque et du volume d’éjection systolique indexé par analyse de l’onde de pouls se fait en utilisant le système PICCO, après calibration par la méthode de thermodilution. La mesure de la variation de pression pulsée est réalisée avec le système PICCO.

### Modalités des mesures respiratoires

Les mesures de pression expiratoire positive totale et de pression de plateau télé-inspiratoire se font en pressant les boutons appropriés du respirateur pour réaliser des pauses télé-expiratoire et télé-inspiratoire par le biais d’une occlusion des valves inspiratoire ou expiratoire, et sont fournies à l’écran.

## Durée prévue de participation des personnes, et description de la chronologie et de la durée de toutes les périodes de la recherche, y compris le suivi, le cas échéant

La durée de participation de chaque patient à partir de son inclusion, sera de 30 minutes environ, distribuée comme suit:

1. Mesures avant remplissage vasculaire 10 minutes

2. Remplissage vasculaire 10 minutes

3. Mesures finales 10 minutes

## Description des règles d’arrêt définitif ou temporaire

### a) de la participation d’une personne à la recherche

Les sujets (ou leurs proches de confiance) pourront retirer leur consentement et demander à sortir de l’étude à n’importe quel moment et quelle qu’en soit la raison. En cas de sortie prématurée, l’investigateur doit en documenter les raisons de façon aussi complète que possible.

L’investigateur pourra interrompre temporairement ou définitivement la participation d’un sujet à l’étude pour toute raison qui servirait au mieux les intérêts du sujet en particulier en cas d’événements indésirables graves.

En cas de retrait de consentement, les données recueillies ne seront pas analysées.

### b) d’une partie ou de la totalité de la recherche

L’étude peut être interrompue prématurément en cas de survenue d’événements indésirables inattendus, graves nécessitant une revue du profil des stratégies médicales étudiées. De même, des événements imprévus ou de nouvelles informations relatives aux stratégies médicales, au vu desquels les objectifs de l'étude ne seront vraisemblablement pas atteints, peuvent amener le promoteur à interrompre prématurément l’étude.

Les Hospices Civils de Lyon se réservent le droit d'interrompre l’étude à tout moment, s'il s'avère que les objectifs d’inclusion ne sont pas atteints.

En cas d’arrêt prématuré de l’étude, l’information sera transmise par le promoteur dans un délai de 15 jours à l’ANSM et au CPP.

## Dispositions mises en œuvre en vue du maintien de l’insu et procédures de levée de l’insu, le cas échéant

Non applicable

## Identification de toutes les données à recueillir directement dans les cahiers d’observation, qui seront considérées comme des données source.

Toutes les données nécessaires à l’analyse seront d’abord documentées dans le dossier médical du sujet, puis retranscrites dans le cahier d’observation (CRF). Aucune donnée source ne sera donc recueillie directement dans le CRF.

Les données suivantes seront relevées à l'inclusion : date de naissance (mois/année), date d’entrée en réanimation, date et heure d’inclusion, date d’obtention des critères diagnostiques de SDRA, sexe, poids mesuré, taille, score IGS II à l'entrée en réanimation, contexte d’admission (médical, post-opératoire ou traumatologie), origine du patient (domicile, service d’urgence, service de court séjour hors réanimation, service de réanimation), étiologie du SDRA, score SOFA le jour de l'inclusion, gaz du sang à l’inclusion (PaO_2_, pH, PaCO_2_, bicarbonates, SaO2), FiO_2_, niveau de PEP externe, volume courant, niveau de PEP totale du système respiratoire, pression plateau du système respiratoire, fréquence respiratoire, traitements parmi les suivants (monoxyde d'azote inhalé, almitrine, curares, épuration extrarénale, administration d’un traitement inotrope, administration d’un traitement vasopresseur, dose de dobutamine, dose de noradrénaline, dose d’adrénaline), cause de la défaillance circulatoire (choc septique, choc hémorragique, choc anaphylactique, syndrome post-arrêt cardiaque, choc cardiogénique, choc anaphylactique), justification du remplissage vasculaire (hypotension artérielle, introduction de traitement vasopresseur, diurèse< 0,5 ml/kg/h, tachycardie> 100/min, marbrures cutanées, hyperlactatémie, baisse du débit cardiaque, saturation veineuse en oxygène inférieure à 65% dans le sang veineux mêlé ou inférieure à 70% en territoire cave supérieur…), taux de lactates artériels, présence d’une fibrillation auriculaire ou d’une autre cause d’arythmie cardiaque.

Les données suivantes seront recueillies pendant la participation à l’étude :

- A l’état de base (BASE)
  - Mesures circulatoires
    - Pression artérielle systolique (PAS), diastolique (PAD) et moyenne (PAM)
    - Fréquence cardiaque (FC)
    - Pression veineuse centrale (PVC)
    - Variation de la pression pulsée (VPP)
    - Index cardiaque (IC) par thermodilution (moyenne de 4 mesures consécutives)
    - Index cardiaque par analyse de l’onde de pouls
    - Volume d’éjection systolique indexé par analyse de l’onde de pouls (VEJI)
    - Volume télédiastolique global indexé (VTDGI)
    - Eau pulmonaire extravasculaire indexée (EPEVI)
- Lors de la mise en position de Trendelenburg pendant 1 minute (TREND)
  - Index cardiaque par analyse de l’onde de pouls (valeur maximale)
  - Volume d’éjection systolique indexé (valeur maximale)
  - Pression veineuse centrale (valeur maximale)
- 1 minute après le retour en position de repos (BASE 2)
  - Pression artérielle systolique, diastolique et moyenne
  - Fréquence cardiaque
  - Pression veineuse centrale
  - Variation de la pression pulsée
  - Index cardiaque par analyse de l’onde de pouls
  - Volume d’éjection systolique indexé par analyse de l’onde de pouls
- Lors d’une augmentation transitoire du volume courant jusqu’à 8 ml/kg de poids prédit par la taille pendant 1 minute (VT8)
  - Pression veineuse centrale
  - Variation de la pression pulsée
  - Index cardiaque par analyse de l’onde de pouls
  - Volume d’éjection systolique indexé par analyse de l’onde de pouls
- 1 minute après le retour en position de repos (BASE 3)
  - Pression artérielle systolique, diastolique et moyenne
  - Fréquence cardiaque
  - Pression veineuse centrale
  - Variation de la pression pulsée
  - Index cardiaque par analyse de l’onde de pouls
  - Volume d’éjection systolique indexé par analyse de l’onde de pouls
- A la fin d’une occlusion télé-expiratoire de 15 secondes (OCC)
  - Pression veineuse centrale
  - Index cardiaque par analyse de l’onde de pouls
  - Volume d’éjection systolique indexé par analyse de l’onde de pouls
- 1 minute après le retour en position de repos (BASE 4)
  - Pression artérielle systolique, diastolique et moyenne
  - Fréquence cardiaque
  - Pression veineuse centrale
  - Variation de la pression pulsée
  - Index cardiaque par analyse de l’onde de pouls
  - Volume d’éjection systolique indexé par analyse de l’onde de pouls
- Après remplissage vasculaire par 500 ml de Ringer lactate en 10 minutes (RV)
  - Pression artérielle systolique, diastolique et moyenne
  - Fréquence cardiaque
  - Pression veineuse centrale
  - Variation de la pression pulsée
  - Index cardiaque par thermodilution
  - Index cardiaque par analyse de l’onde de pouls
  - Volume d’éjection systolique indexé par analyse de l’onde de pouls
  - Volume télédiastolique global indexé
  - Eau pulmonaire extravasculaire indexée (EPEVI)

Les données relatives à la sécurité seront collectées de façon prospective pour documenter les effets délétères éventuels de la position de Trendelenburg : chute de la pression artérielle systolique > 30 mm Hg, augmentation de la fréquence cardiaque > 10%, diminution de la saturation artérielle transcutanée en oxygène en dessous de 88%, survenue d’un trouble du rythme cardiaque, autre…

# Sélection et exclusion des personnes de la recherche

Les patients seront éligibles pour l'étude s'ils présentent l'ensemble des critères d'inclusion et de non inclusion suivants :

## Critères d’inclusion

- Patient sous ventilation mécanique, en décubitus ventral, avec SDRA défini par les 4 critères suivants ^1^:
  - insuffisance respiratoire aiguë survenant dans la semaine suivant une agression pulmonaire,
  - opacités pulmonaires bilatérales non expliquées par des épanchements pleuraux, des atélectasies ou des nodules
  - une hypoxémie définie par un rapport PaO2/FiO2 < 300 sous PEEP ≥ 5 cm d’H2O
  - non expliquée par une insuffisance cardiaque ou une hypervolémie
- Indication de remplissage vasculaire posée par le clinicien en charge du patient
- Patient en décubitus ventral sous ventilation mécanique en ventilation assistée contrôlée
- Absence d’effort inspiratoire détectable lors des occlusions télé-expiratoires et télé-inspiratoires de 3 secondes
- Cathéter veineux central implanté en territoire cave supérieur fonctionnel
- Cathéter artériel de type Picco implanté en position fémorale
- Patient affilié à un régime de sécurité sociale
- Consentement signé par un proche.

Dans l’hypothèse où le proche du patient n’est pas joignable, et dans la mesure où l’indication de remplissage vasculaire traduit une situation d’urgence hémodynamique, le malade pourra être inclus dans le protocole selon une clause d’urgence. Le consentement de ses proches sera ensuite recherché.

## Critères d'exclusion

- Age inférieur à 18 ans
- Contre-indication à la mise en position de Trendelenburg (hypertension intracrânienne documentée par la mesure invasive de la pression intracrânienne ou pathologie neurologique de moins de 1 mois à risque d’œdème cérébral et d’hypertension intracrânienne)
- Obstruction cave inférieure
- Patient précédemment inclus dans le même protocole de recherche
- Patient sous mesure de protection administrative et judiciaire

## Procédure d’arrêt prématuré de la recherche ou d’exclusion pour une personne de la recherche et procédure de suivi de la personne

Le patient sort de l’étude en cas de survenue d’un évènement indésirable grave en cours de procédure. Dans ce cas, le patient sera exclu de l’analyse et celui-ci sera remplacé par un patient supplémentaire pour obtenir le nombre de sujet nécessaire requis.

# Traitement administré aux personnes qui se prêtent à la recherche

La participation au protocole n'implique l'utilisation d'aucun médicament autre que ceux habituellement utilisés dans le cadre du soin des patients atteints de SDRA. Il n'y a aucun médicament ou traitement interdit dans le cadre de ce protocole.

# Évaluation de l’efficacité

## Calendrier prévus pour mesurer, recueillir et analyser les paramètres d'évaluation de l’efficacité.

Les paramètres d'efficacité seront analysés secondairement, que ce soit les données d'imagerie ou les paramètres de mécanique respiratoire. Ils seront analysés par un co-investigateur en insu vis à vis des conditions expérimentales de mesure.

Les données individuelles ne seront regroupées que lors de l'analyse finale après inclusion du dernier patient dans l'étude. Aucune analyse intermédiaire n'est prévue vu le faible effectif de malades.

# Évaluation de la sécurité

## Définitions

### Evénement indésirable

Toute manifestation nocive survenant chez une personne qui se prête à une recherche biomédicale que cette manifestation soit liée ou non à la recherche.

### Evénement indésirable grave (EIG)

Un événement grave est un événement :

- dont l'évolution est fatale ; ou

- qui est met en danger la vie de la personne qui se prête à la recherche ; ou

- qui entraîne une incapacité ou un handicap important ou durable ; ou

- qui provoque une hospitalisation ou une prolongation d'hospitalisation ; ou

- qui a pour conséquence une anomalie ou une malformation congénitale ; ou

- tout autre événement médicalement pertinent selon le jugement de l’investigateur ;

## Modalités de détection et de recueil des événements indésirables

Tous les événements indésirables doivent être recherchés, rapportés et enregistrés, traités et évalués de la première visite (inclusion J0) jusqu’à à la fin de l’étude et jusqu’à leur résolution. Les événements indésirables sont recueillis :

- lors des examens cliniques, biologiques ou autres prévus et par un interrogatoire systématique par l’investigateur ;

- par notification spontanée par les participants, qui seront informés de la nécessité de contacter le médecin investigateur en cas d’événement indésirable.

Tous les événements indésirables seront notés sur les formulaires de recueil des événements indésirables du cahier d’observation. Chaque événement indésirable observé sera consigné individuellement. L’intensité des événements indésirables sera déterminée de la façon suivante :

- légère (grade 1) : pas d’interférence sur l’activité au quotidienne du patient ;

- modérée (grade 2) : interférence modérée sur l’activité quotidienne du patient mais encore acceptable ;

- sévère (grade 3) : interférence importante sur l’activité quotidienne du patient et inacceptable ;

- menace du pronostic vital (grade 4) ;

- décès (grade 5).

Tous les évènements indésirables doivent être gradés.

## Responsabilités de l’investigateur

### Déclaration des EIG

L’investigateur évalue chaque événement indésirable au regard de sa gravité.

L’investigateur doit notifier par FAX (04 72 11 51 90) au promoteur, sans délai, à compter du jour où il en a connaissance, tous les événements indésirables graves survenus dans l’essai, à l’exception de ceux qui sont recensés dans le protocole comme ne nécessitant pas une notification immédiate. Cette notification initiale fait l’objet d’un rapport écrit et doit être suivie si nécessaire par un ou des rapport(s) complémentaire(s) écrit(s) détaillé(s) détaillé(s) dans les 8 jours suivant la première déclaration.

L’investigateur doit documenter au mieux l’événement (grâce aux copies des résultats de laboratoires ou des comptes rendus d’examens ou d’hospitalisation renseignant l’événement grave, y compris les résultats négatifs pertinents, sans omettre de rendre ces documents anonymes et d’inscrire le numéro et le code du patient), le diagnostic médical et établir un lien de causalité entre l’événement indésirable grave et le(s) produit(s) expérimental(aux) et/ou la recherche.

La déclaration est transmise au promoteur à l'aide du formulaire de déclaration d’événement indésirable grave daté et signé.

L’investigateur doit suivre le patient ayant présenté un EIG jusqu’à sa résolution, une stabilisation à un niveau jugé acceptable par l’investigateur ou le retour à l’état antérieur, même si le patient est sorti de l’essai et informer le promoteur par fax au 04 72 11 51 90 à l’aide du formulaire (cocher la case:  suivi).

### Evaluation de la causalité

L’investigateur doit évaluer le lien de causalité des événements indésirables avec la recherche. Le lien de causalité est binaire (relié / non relié).

## Responsabilités du promoteur

Le promoteur déclarera tous les effets indésirables graves inattendus, les faits nouveaux de sécurité et établira un rapport annuel de sécurité conformément à la Loi du 9 Août 2004.

## Constitution d’un comité de surveillance indépendant

Dans la mesure où il n’est attendu aucun évènement indésirable grave lié à la participation des patients dans cette étude, nous ne souhaitons pas mettre en place de comité de surveillance indépendant.

# Statistiques

## Description des méthodes statistiques prévues, y compris du calendrier des analyses intermédiaires prévues

L'analyse statistique sera réalisée avec le logiciel R ^31^, par l’un des co-investigateurs (Pr Claude Guérin).

### Analyse intermédiaire

Il n'y a pas d'analyse statistique intermédiaire prévue au vu du faible effectif de l'étude.

### Caractéristiques de la population lors de l’inclusion

Répartition en fréquence absolue et relative pour les variables qualitatives.

Calcul des moyennes, déviation standard, médiane, 1^er^ et 3^ème^ quartiles, pour les variables quantitatives.

### Analyse des critères de jugement

La normalité des données sera évaluée avec un test de Shapiro-Wilk. Les patients seront classés comme précharge-dépendants si leur index cardiaque augmente de plus de 15% après remplissage vasculaire en comparaison avec la mesure à l’état basal (Base 1).

Les données obtenues à base 1 et RV seront comparées par un test t pairé ou un test de Wilcoxon. Les données obtenues à base 2, 3 et 4 ne seront pas testées et sont utilisées pour vérifier le retour des variables mesurées à l’état de base.

La sensibilité, spécificité et les valeurs prédictives positives et négatives seront exprimées sous la forme d’une moyenne et d’un intervalle de confiance à 95%, pour chacun des 4 paramètres suivants (variabilité de la pression pulsée à l’état de base, variabilité de la pression pulsée après augmentation du volume courant, variation de l’index cardiaque pendant l’occlusion télé-expiratoire, variation de l’index cardiaque pendant la mise en Trendelenburg). Les courbes ROC seront construites pour évaluer la performance de chacun de ces 4 paramètres pour prédire la précharge dépendance. Les aires sous les courbes ROC pour chacun de ces paramètres seront comparées en utilisant le test de Hanley-McNeil.

## Nombre prévu de personnes à inclure dans la recherche, et nombre prévu de personnes dans chaque lieu de recherches avec sa justification statistique

Le nombre de sujet nécessaire a été évalué de la façon suivante. L’objectif fixé est de trouver un paramètre avec une aire sous la courbe ROC (ASC) de 0,8 ; une performance considérée comme cliniquement acceptable pour évaluer la précharge-dépendance. En faisant l’hypothèse d’une prévalence de la précharge-dépendance de 50%, et en visant un intervalle de confiance à 95% de l’aire sous la courbe ROC de ± 0,15, le nombre de sujets nécessaires a été calculé à 33 ^27^. Il est donc prévu d’inclure 33 patients.

## Degré de signification statistique prévu

Le degré de significativité statistique a été fixé inférieur au seuil de 5%.

## Critères statistiques d’arrêt de la recherche

Aucun. La recherche est conduite jusqu’au dernier malade prévu.

## Méthode de prise en compte des données manquantes, inutilisées ou non valides

On cherchera au maximum à éviter les données manquantes. Les données non valides seront évitées par les précautions suivantes : le contrôle de qualité des données sera réalisé en permanence pendant l’acquisition des mesures hémodynamiques et respiratoires. La position des capteurs de pression sera vérifiée régulièrement. Le contrôle de qualité des courbes de pressions artérielle et veineuse centrale sera réalisé par le contrôle du zéro de pression en début de protocole, et un flush régulier du cathéter. L’absence d’effort inspiratoire sur les courbes de pression et de débit des voies aériennes sera contrôlé pendant l’occlusion télé-expiratoire sur l’écran du respirateur

## Gestion des modifications apportées au plan d’analyse de la stratégie initiale

Un plan d’analyse statistique détaillé sera rédigé avant le gel de la base des données. Il tiendra compte de toute modification du protocole ou de tout événement inattendu survenu au cours de l’étude et ayant un impact sur les analyses présentées ci-dessus. Les analyses planifiées pourront être complétées en cohérence avec les objectifs de l’étude.

## Toute modification apportée par la suite au plan d’analyse statistique devra être justifiée et donnera lieu à une nouvelle version du document. Ces déviations au plan d’analyse seront reportées dans le rapport final de l’étude. L’ensemble des documents sera conservé dans le dossier de l’étude.Choix des personnes à inclure dans les analyses

Tous les malades chez qui la mesure des critères de jugement principaux a été possible.

# Droit d'accès aux données et documents source.

## Accès aux données :

Conformément aux BPC :

- le promoteur est chargé d’obtenir l’accord de l’ensemble des parties impliquées dans la recherche afin de garantir l’accès direct à tous les lieux de déroulement de la recherche, aux données sources, aux documents sources et aux rapports dans un but de contrôle de qualité et d’audit par le promoteur ;

## - les investigateurs mettront à disposition des personnes chargées du suivi, du contrôle de qualité ou de l'audit de la recherche biomédicale, les documents et données individuelles strictement nécessaires à ce contrôle, conformément aux dispositions législatives et réglementaires en vigueur (articles L.1121-3 et R.5121-13 du code de la santé publique).Documents sources

Les documents sources sont définis comme tout document ou objet original permettant de prouver l'existence ou l'exactitude d'une donnée ou d'un fait enregistrés au cours de l’étude clinique. Ils seront conservés pendant 15 ans par l'investigateur ou par l'hôpital s'il s'agit d'un dossier médical hospitalier.

Le dossier médical, les compte-rendu d’analyses biologiques, les enregistrements des différentes mesures constituent donc des documents sources.

## Confidentialité des données

Conformément aux dispositions concernant la confidentialité des données auxquelles ont accès les personnes chargées du contrôle de qualité d’une recherche biomédicale (article L.1121-3 du code de la santé publique), conformément aux dispositions relatives à la confidentialité des informations concernant notamment les essais, les personnes qui s'y prêtent et les résultats obtenus (article R. 5121-13 du code de la santé publique), les personnes ayant un accès direct aux données prendront toutes les précautions nécessaires en vue d'assurer la confidentialité des informations relatives aux essais, aux personnes qui s'y prêtent et notamment en ce qui concerne leur identité ainsi qu’aux résultats obtenus.

Ces personnes, au même titre que les investigateurs eux-mêmes, sont soumises au secret professionnel (selon les conditions définies par les articles 226-13 et 226-14 du code pénal).

Pendant la recherche biomédicale ou à son issue, les données recueillies sur les personnes qui s’y prêtent et transmises au promoteur par les investigateurs (ou tous autres intervenants spécialisés) seront rendues anonymes.

Elles ne doivent en aucun cas faire apparaître en clair les noms des personnes concernées ni leur adresse.

Seules la première lettre du nom du sujet et la première lettre de son prénom seront enregistrées, accompagnées d’un numéro codé propre à l’étude indiquant l’ordre d’inclusion des sujets.

Le promoteur s’assurera que chaque personne qui se prête à la recherche a donné son accord par écrit pour l’accès aux données individuelles la concernant et strictement nécessaires au contrôle de qualité de la recherche.

# Contrôle et assurance de la qualité

Un Attaché de Recherche Clinique (ARC) mandaté par le promoteur s’assurera de la bonne réalisation de l’étude, du recueil des données générées par écrit, de leur documentation, enregistrement et rapport, en accord avec les Procédures Opératoires Standards mises en application au sein de la DRCI des Hospices Civils de Lyon et conformément aux Bonnes Pratiques Cliniques ainsi qu’aux dispositions législatives et réglementaires en vigueur.

L'investigateur et les membres de son équipe acceptent de se rendre disponibles lors des visites de Contrôle de Qualité effectuées à intervalles réguliers par l’Attaché de Recherche Clinique. Lors de ces visites, les éléments suivant pourront être revus :

- consentement éclairé
- respect du protocole de l'étude et des procédures qui y sont définies
- qualité des données recueillies dans le cahier d'observation : exactitude, données manquantes, cohérence des données avec les documents sources
- déclaration des événements indésirables graves.

D’autre part, Les investigateurs s’engagent à accepter les audits d’assurance qualité effectués par le promoteur ainsi que les inspections effectuées par les Autorités Compétentes. Toutes les données, tous les documents et rapports peuvent faire l'objet d'audits et d'inspections réglementaires sans que puisse être opposé le secret médical.

# Considérations éthiques.

Il s’agit d’une recherche biomédicale. L’étude sera conduite conformément au protocole, aux lignes directives des Bonnes Pratiques Cliniques françaises et européennes, à la déclaration d’Helsinki dans sa dernière version (Séoul 2008) ainsi qu’aux recommandations de l’ICH (International Conference on Harmonisation - Guideline for Good Clinical Practice) et à la loi n° 2004-806 du Code de la santé Publique, relative aux recherches biomédicales.

Conformément aux dispositions de la loi n° 2004-806 du Code de la Santé Publique, le protocole sera soumis à l’avis du CPP Sud-Est IV et à l’autorisation de l'ANSM (autorité compétente).

## Autorités compétentes

Le protocole, la notice d'information et le formulaire de consentement de l'étude seront soumis pour avis au Comité de Protection des Personnes Sud-Est IV.

La notification de l'avis favorable du CPP sera transmise au promoteur de l'étude et à l’ANSM. Une demande d’autorisation d’étude sera également adressée par le promoteur à l'ANSM.

Le promoteur s’engage à ce que le démarrage de l’étude ne se fasse qu’après obtention de l’avis favorable du CPP et de l’autorisation d’étude de l’ANSM.

## Modifications substantielles

En cas de modification substantielle apportée au protocole par l’investigateur, elle sera approuvée par le promoteur. Ce dernier devra obtenir préalablement à sa mise en œuvre un avis favorable du CPP et une autorisation de l’ANSM dans le cadre de leurs compétences respectives. Un nouveau consentement des personnes participant à la recherche sera recueilli si nécessaire.

## Information du patient et formulaire de consentement écrit

Les proches des patients seront informés de façon complète et loyale, en des termes compréhensibles, des objectifs et des contraintes de l'étude, des risques éventuels encourus, des mesures de surveillance et de sécurité nécessaires, de leurs droits de refuser de participer à l'étude ou de la possibilité de se rétracter à tout moment.

Toutes ces informations figurent sur un formulaire d’information et de consentement remis au proche du patient. Le consentement libre, éclairé et écrit du proche du patient sera recueilli par l’investigateur, ou un médecin qui le représente avant l’inclusion définitive dans l’étude. Une copie du formulaire d'information et de consentement signé par les deux parties sera remise au patient, l’investigateur en conservera l’original.

Le consentement d’utiliser les données sera demandé au patient dès qu’il sera en mesure de le donner.

Dans l’hypothèse où les proches du patient ou la personne de confiance n’est pas joignable, et dans la mesure où l’indication de remplissage vasculaire traduit une situation d’urgence hémodynamique, le malade pourra être inclus dans le protocole selon une clause d’urgence selon l’article L1122-1-2 du code de la santé publique. Le consentement des proches ou de la personne de confiance sera ensuite recherché. La procédure de recherche, d'identification, de sollicitation de la personne de confiance, et la traçabilité éventuelle de son absence seront identifiées dans le dossier médical du patient. Le consentement d’utiliser les données sera demandé au patient dès qu’il sera en mesure de le donner.

## Période d’exclusion

Il n’est prévu aucune période d’exclusion à l’issue de la recherche. Le patient ne peut pas participer à un autre projet de recherche biomédicale au cours de l’étude.

## Prise en charge relative à la recherche

En dehors de la position de Trendelenburg, l’ensemble des examens ou mesures est réalisé de manière habituelle.

## Indemnisation des sujets

Il n’est pas prévu d’indemnisation des participants à l’étude.

## Inscription au fichier national des personnes se prêtant à une recherche biomédicale

L’objet de la recherche est en rapport direct avec l’état pathologique des patients. Il n’est donc pas prévu d’inscription sur le fichier national des personnes se prêtant à une recherche.

# Traitement des données et conservation des documents et des données relatives à la recherche.

## Cahier d’observation

Le cahier d'observation ne comportera que les données nécessaires à l'analyse en vue de publication. Les autres données relatives au patient et nécessaires à son suivi en dehors de l'étude, seront colligées dans son dossier médical.

Toutes les informations requises par le protocole doivent être consignées dans les cahiers d’observation. Les données devront être recueillies au fur et à mesure qu'elles sont obtenues, et enregistrées dans ces cahiers de façon explicite. Chaque donnée manquante devra être codée.

## Saisie et analyse des données

La saisie et l'analyse des données seront réalisées par Jean-Christophe Richard.

## CNIL

Pour cette étude, conformément à la loi relative à l’Informatique, aux fichiers et aux libertés, l’investigateur principal et le promoteur s’engagent à respecter la méthodologie de référence (MR001) de la Commission Nationale de l’Informatique et des Libertés (CNIL).

## Archivage

Les documents suivants seront archivés par le nom de l’étude dans les locaux du service de réanimation médicale des Hospices Civils de Lyon (Croix-Rousse) jusqu’à la fin de la période d’utilité pratique.

Ces documents sont :

- Protocole et annexes, amendements éventuels,
- Formulaires d’information et consentements originaux signés
- Données individuelles (copies authentifiées de données brutes)
- Documents de suivi
- Analyses statistiques
- Rapport final de l’étude

A l’issue de la période d’utilité pratique, l’ensemble des documents à archiver, tels que définis dans la procédure de « classement et archivage des documents liés aux recherches biomédicales » des Hospices Civils de Lyon, sera transféré sur le site d’archivage (Service Central des Archives – Hospices Civils de Lyon) et sera placé sous la responsabilité du Promoteur pendant 15 ans après la fin de l’étude.

Aucun déplacement ou destruction ne pourra être effectué sans l’accord du promoteur. Au terme des 15 ans, le promoteur sera consulté pour destruction. Toutes les données, tous les documents et rapports pourront faire l’objet d’audit ou d’inspection.

# Financement et assurance

## Financement

Cette étude ne requiert pas de financement.

## Assurance

Conformément aux dispositions de l’article L 209.7 du code de la santé publique, le promoteur de l’essai a souscrit une assurance responsabilité civile auprès de la Société Hospitalière d’Assurance Mutuelle, 18 rue Edouard Rochet, 69008 Lyon, sous le numéro de contrat 134823.

# Règles relatives à la publication.

Nous chercherons à publier la recherche au plus haut niveau possible, en langue anglaise, par exemple dans la revue Intensive Care Medicine (impact facteur 5,055 en 2009). Les HCL seront cités comme promoteur de l’étude.

# Calendrier prévisionnel

Décembre 2012 : protocole soumis à la promotion des Hospices Civils de Lyon

Février 2013 : obtention de l'accord de promotion des Hospices Civils de Lyon

Février 2013 : protocole soumis au CPP de l’inter Région Sud-Est IV et à l’autorité compétente par le promoteur

Juillet 2013 : accord du CPP de l’inter Région Sud-Est IV et de l’autorité compétente

Octobre 2013 : début des inclusions

Octobre 2017 : fin des inclusions

Octobre 2017 à décembre 2017 : analyse des données

Une demande de prolongation de 2 ans a été demandée en raison d’un rythme d’inclusion inférieur à la prévision (23 patients inclus après 2 ans).

# Bibliographie

1. Ferguson ND, Fan E, Camporota L, et al. The Berlin definition of ARDS: an expanded rationale, justification, and supplementary material. Intensive Care Med 2012;38(10):1573–82.

2. Vincent JL, Weil MH. Fluid challenge revisited. Crit Care Med 2006;34(5):1333–7.

3. Marik PE, Cavallazzi R, Vasu T, Hirani A. Dynamic changes in arterial waveform derived variables and fluid responsiveness in mechanically ventilated patients: a systematic review of the literature. Crit Care Med 2009;37(9):2642–7.

4. Boyd JH, Forbes J, Nakada T, Walley KR, Russell JA. Fluid resuscitation in septic shock: a positive fluid balance and elevated central venous pressure are associated with increased mortality. Crit Care Med 2011;39(2):259–65.

5. Maitland K, Kiguli S, Opoka RO, et al. Mortality after fluid bolus in African children with severe infection. N Engl J Med 2011;364(26):2483–95.

6. Wiedemann HP, Wheeler AP, Bernard GR, et al. Comparison of two fluid-management strategies in acute lung injury. N Engl J Med 2006;354(24):2564–75.

7. Sakr Y, Vincent JL, Reinhart K, et al. High tidal volume and positive fluid balance are associated with worse outcome in acute lung injury. Chest 2005;128(5):3098–108.

8. Teboul J-L. Recommandations d’experts de la SRLF: « Indicateurs du remplissage vasculaire au cours de l’insuffisance circulatoire ». Réanimation 2004;13(4):255–63.

9. Michard F, Teboul JL. Predicting fluid responsiveness in ICU patients: a critical analysis of the evidence. Chest 2002;121(6):2000–8.

10. Michard F, Boussat S, Chemla D, et al. Relation between respiratory changes in arterial pulse pressure and fluid responsiveness in septic patients with acute circulatory failure. Am J Respir Crit Care Med 2000;162(1):134–8.

11. De Backer D, Heenen S, Piagnerelli M, Koch M, Vincent JL. Pulse pressure variations to predict fluid responsiveness: influence of tidal volume. Intensive Care Med 2005;31(4):517–23.

12. Reuter DA, Bayerlein J, Goepfert MS, et al. Influence of tidal volume on left ventricular stroke volume variation measured by pulse contour analysis in mechanically ventilated patients. Intensive Care Med 2003;29(3):476–80.

13. Ventilation with lower tidal volumes as compared with traditional tidal volumes for acute lung injury and the acute respiratory distress syndrome. N Engl J Med 2000;342(18):1301–8.

14. Richard JC, Girault C, Leteurtre S, Leclerc F, SRLF G d’expert de la. Ventilatory management of acute respiratory distress syndrome (ARDS) in adult patients and children (excluding neonates). Réanimation 2005;14(7):323–32.

15. Wong DH, Tremper KK, Zaccari J, Hajduczek J, Konchigeri HN, Hufstedler SM. Acute cardiovascular response to passive leg raising. Crit Care Med 1988;16(2):123–5.

16. Boulain T, Achard JM, Teboul JL, Richard C, Perrotin D, Ginies G. Changes in BP induced by passive leg raising predict response to fluid loading in critically ill patients. Chest 2002;121(4):1245–52.

17. Cavallaro F, Sandroni C, Marano C, et al. Diagnostic accuracy of passive leg raising for prediction of fluid responsiveness in adults: systematic review and meta-analysis of clinical studies. Intensive Care Med 2010;36(9):1475–83.

18. Abroug F, Ouanes-Besbes L, Elatrous S, Brochard L. The effect of prone positioning in acute respiratory distress syndrome or acute lung injury: a meta-analysis. Areas of uncertainty and recommendations for research. Intensive Care Med 2008;34(6):1002–11.

19. Alsaghir AH, Martin CM. Effect of prone positioning in patients with acute respiratory distress syndrome: a meta-analysis. Crit Care Med 2008;36(2):603–9.

20. Sud S, Sud M, Friedrich JO, Adhikari NK. Effect of mechanical ventilation in the prone position on clinical outcomes in patients with acute hypoxemic respiratory failure: a systematic review and meta-analysis. CMAJ 2008;178(9):1153–61.

21. Kopterides P, Siempos I, Armaganidis A. Prone positioning in hypoxemic respiratory failure: meta-analysis of randomized controlled trials. J Crit Care 2009;24(1):89–100.

22. Gattinoni L, Carlesso E, Taccone P, Polli F, Guerin C, Mancebo J. Prone positioning improves survival in severe ARDS: a pathophysiologic review and individual patient meta-analysis. Minerva Anestesiol 2010;76(6):448–54.

23. Sud S, Friedrich JO, Taccone P, et al. Prone ventilation reduces mortality in patients with acute respiratory failure and severe hypoxemia: systematic review and meta-analysis. Intensive Care Med 2010;36(4):585–99.

24. Abroug F, Ouanes-Besbes L, Dachraoui F, Ouanes I, Brochard L. An updated study-level meta-analysis of randomised controlled trials on proning in ARDS and acute lung injury. Crit Care 2011;15(1):R6.

25. Guérin C, Reignier J, Richard J-C, et al. Prone positioning in severe acute respiratory distress syndrome. N Engl J Med 2013;368(23):2159–68.

26. Monnet X, Osman D, Ridel C, Lamia B, Richard C, Teboul JL. Predicting volume responsiveness by using the end-expiratory occlusion in mechanically ventilated intensive care unit patients. Crit Care Med 2009;37(3):951–6.

27. Obuchowski NA. Sample size calculations in studies of test accuracy. Stat Methods Med Res 1998;7(4):371–92.
