# Supplementary material for: Change in cardiac output during Trendelenburg maneuver is a reliable predictor of fluid responsiveness in patients with acute respiratory distress syndrome in the prone position under protective ventilation
Source: Crit Care. 2017 Dec 5;21:295. doi: 10.1186/s13054-017-1881-0 (PMC5718075; doi:10.1186/s13054-017-1881-0)
Supplement: Supplementary file 2 — Protocol description. (DOCX 71 kb) [file 13054_2017_1881_MOESM2_ESM.docx]

**Figure S1**. Protocol description.

BA = bed angulation; EEO = end-expiratory occlusion, VT = tidal volume.
