# Supplementary figures and images for: Change in cardiac output during Trendelenburg maneuver is a reliable predictor of fluid responsiveness in patients with acute respiratory distress syndrome in the prone position under protective ventilation
Source: Crit Care. 2017 Dec 5;21:295. doi: 10.1186/s13054-017-1881-0 (PMC5718075; doi:10.1186/s13054-017-1881-0)

**Figure S2**. Study flow chart.


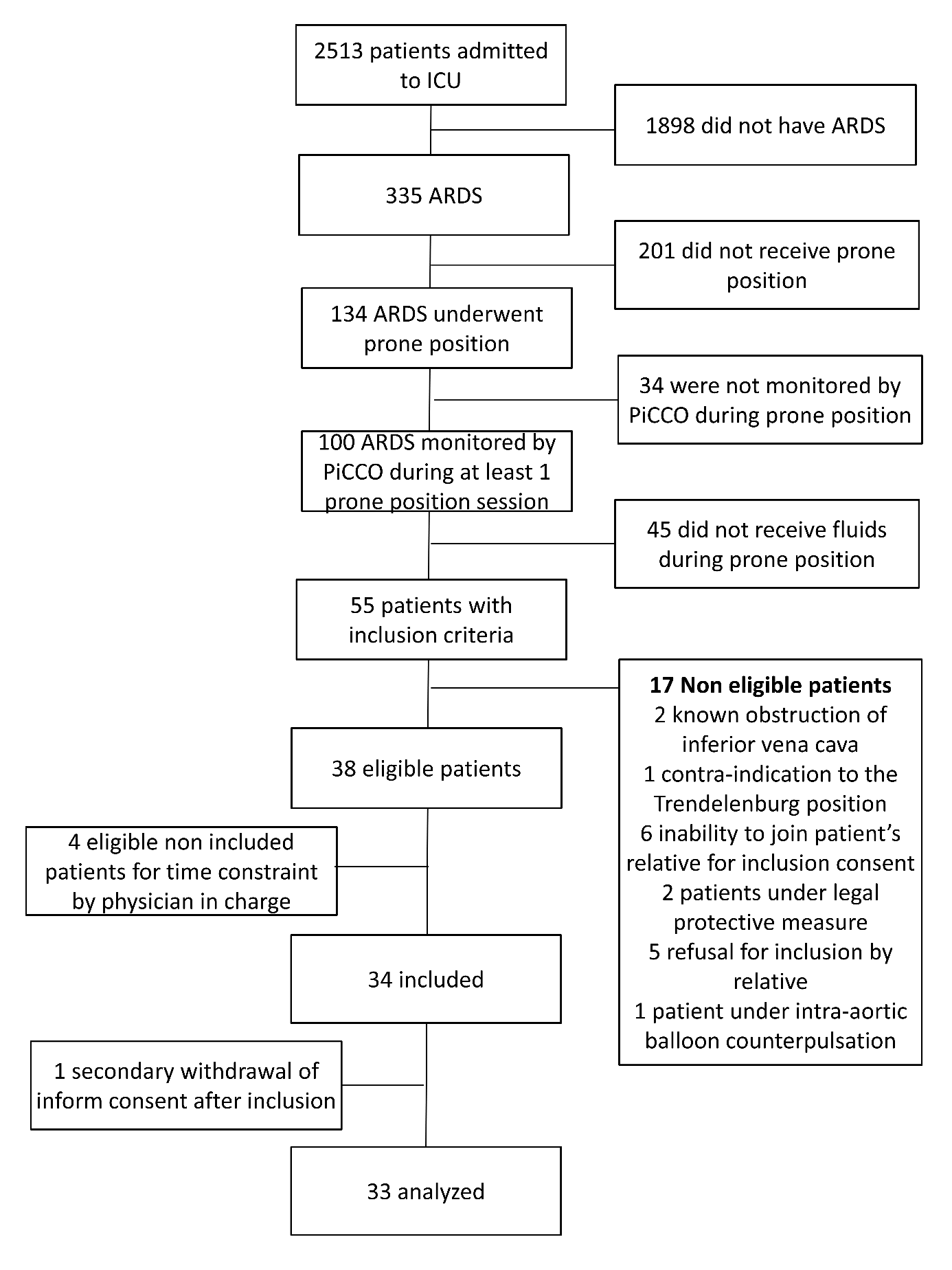


ARDS = acute respiratory distress syndrome; ICU = intensive care unit.

Supplement: Supplementary file 3 — Study flow chart. (DOCX 397 kb) [file 13054_2017_1881_MOESM3_ESM.docx]
