# Supplementary material for: Change in cardiac output during Trendelenburg maneuver is a reliable predictor of fluid responsiveness in patients with acute respiratory distress syndrome in the prone position under protective ventilation
Source: Crit Care. 2017 Dec 5;21:295. doi: 10.1186/s13054-017-1881-0 (PMC5718075; doi:10.1186/s13054-017-1881-0)
Supplement: Supplementary file 4 — Change in transpulmonary thermodilution-cardiac index by volume expansion (ΔCIVE) as function of response to five diagnostic tests. (DOCX 314 kb) [file 13054_2017_1881_MOESM4_ESM.docx]

**Figure S3**. Change in transpulmonary thermodilution-cardiac index by volume expansion (ΔCI_VE_) as function of response to 5 diagnostic tests.

Response to each test below the lower or above the higher border of the gray zone (area of uncertainty of optimal cut-off points) were considered negative and positive, respectively. Response to test within the gray zone were considered inconclusive. Some box are lacking for some tests since no patient belonged to these groups.


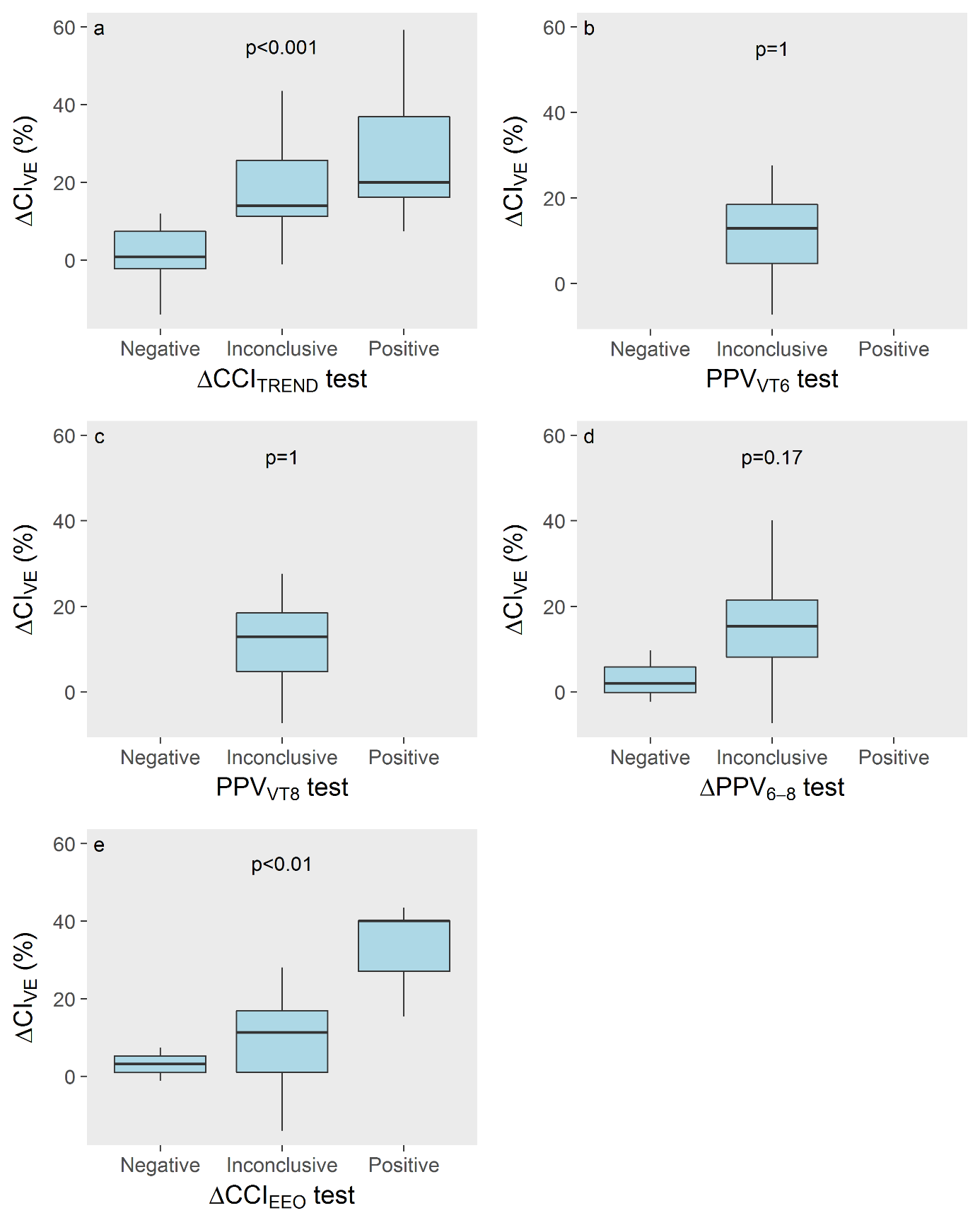


ΔCCI_TREND_ = change in continuous cardiac index during the Trendelenburg maneuver; ΔCCI_EEO_ = change in continuous cardiac index during end-expiratory occlusion; ΔCI_VE_ = change in transpulmonary thermodilution-cardiac index by volume expansion; PPV_VT6_ = pulse pressure variation during ventilation with 6 ml.kg^-1^ predicted body weight tidal volume; PPV_VT8_ = pulse pressure variation during ventilation with 8 ml.kg^-1^ predicted body weight tidal volume; ΔPPV_6-8_ = change in pulse pressure variation between ventilation with 6 and 8 ml.kg^-1^ predicted body weight tidal volume.
