# Supplementary material for: Change in cardiac output during Trendelenburg maneuver is a reliable predictor of fluid responsiveness in patients with acute respiratory distress syndrome in the prone position under protective ventilation
Source: Crit Care. 2017 Dec 5;21:295. doi: 10.1186/s13054-017-1881-0 (PMC5718075; doi:10.1186/s13054-017-1881-0)
Supplement: Supplementary file 5 — Diagnostic performance of end-expiratory occlusion to predict fluid responsiveness as a function of change in CVP (ΔCVP) during the test as compared to baseline. (DOCX 14 kb) [file 13054_2017_1881_MOESM5_ESM.docx]

**Table S1**. Diagnostic performance of end-expiratory occlusion to predict fluid responsiveness as a function of change in CVP (ΔCVP) during the test as compared to baseline.

| Subgroups | Number of patients | Number of fluid responders | AUC  [CI_95%_] | p value vs. an AUC of 0.5 | Optimal threshold | Sensitivity [CI_95%_] | Specificity [CI_95%_] | PLR | NLR |
| --- | --- | --- | --- | --- | --- | --- | --- | --- | --- |
| ΔCVP < 0 | 19 | 10 (53%) | 0.45  [0.18-0.72] | 0.73 | 6% | 70%  [40%-100%] | 44%  [11%-78%] | 1.25 | 0.68 |
| ΔCVP ≥ 0 | 14 | 5 (36%) | 0.89  [0.70-1.00] | 0.02 | 6% | 80%  [40%-100%] | 89%  [67%-89%] | 7.27 | 0.22 |

AUC = area under ROC curve; CI_95%_ = 95% confidence interval; NLR = negative likehood ratio; PLR = positive likehood ratio.
