# Supplementary material for: Change in cardiac output during Trendelenburg maneuver is a reliable predictor of fluid responsiveness in patients with acute respiratory distress syndrome in the prone position under protective ventilation
Source: Crit Care. 2017 Dec 5;21:295. doi: 10.1186/s13054-017-1881-0 (PMC5718075; doi:10.1186/s13054-017-1881-0)
Supplement: Supplementary file 6 — Receiver operating characteristics curves of end-expiratory occlusion to predict fluid responsiveness as a function of change in CVP (ΔCVP) during the test as compared to baseline. (DOCX 67 kb) [file 13054_2017_1881_MOESM6_ESM.docx]

**Figure S4**. Receiver operating characteristics curves of end-expiratory occlusion to predict fluid responsiveness as a function of change in CVP (ΔCVP) during the test as compared to baseline.
